# Supplementary material for: Multiple mechanisms of termination modulate the dynamics of RNAPI transcription
Source: Cell Rep. Author manuscript; Available in PMC 2026 Feb 3. (PMC7618708; doi:10.1016/j.celrep.2025.115325)
Supplement: Supplementary Material [file EMS212091-supplement-Supplementary_Material.zip › 1-s2.0-S2211124725000968-mmc1.pdf]

Cell Reports, Volume *44*

Supplemental information

Multiple mechanisms of termination modulate the dynamics of RNAPI transcription

Elisabeth Petfalski, Marie-Luise Winz, Katarzyna Grelewska-Nowotko, Tomasz W. Turowski, and David Tollervey

SUPPLEMENTARY MATERIAL

Figure S1. Distribution of Rpa190 along the rDNA (related to Figure 1)
Figure S2. RNAPI pause sites in transcription termination region (related to Figure 2)
Figure S3. Schematic of *in vitro* termination assay (related to Figure 3)
Figure S4. Rat1 and poly(A) tails are found at sites of slowed RNAPI elongation (related to Figure 4)
Figure S5. RNAPI and Top2 distributions along the 5’ ETS (related to Figure 5)
Figure S6. Development of premature termination P_{PT} function (related to Figure 6)

Table S1. Oligonucleotides used for strain construction
Table S2. Oligonucleotides used for *in vitro* assay

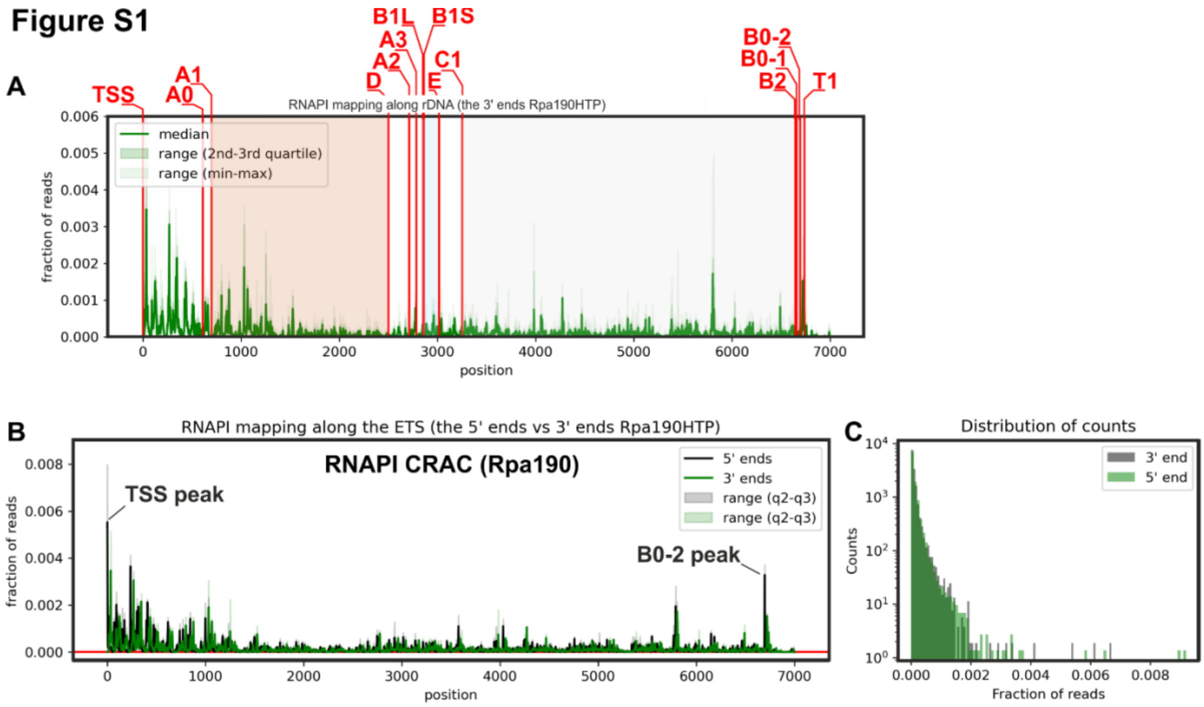

Figure S1. Distribution of Rpa190 along the rDNA (related to Figure 1). Data are represented as median (n=6).
A: Rpa190 distribution over rDNA transcription unit with marked processing sites.
B: RNAPI Rpa190-HTP CRAC mapping of the 5’ ends (black) and the 3’ ends (green) of reads. Note that substantial differences between the 5’ and 3’ ends are visible for the very 5’ end at the transcription start site (TSS peak) and within the 3’ ETS, at the B0-2 cleavage site (B0-2 peak).
C: 5’ ends of RNAPI CRAC reads are less distributed relative to 3’ ends.

Figure S2

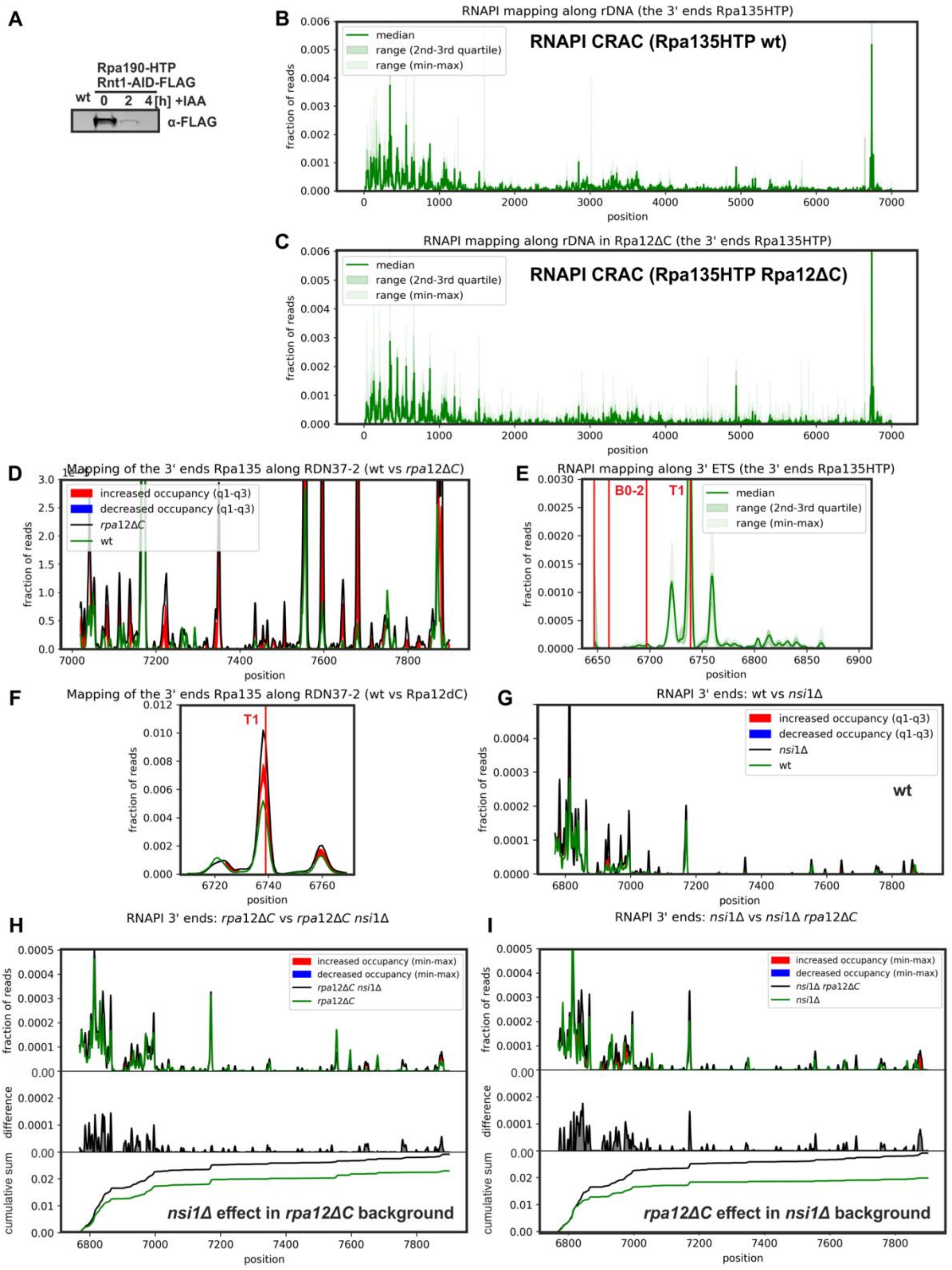

Figure S2. RNAPI pause sites in transcription termination region (related to Figure 2). Data are represented as median (Rpa135-HTP n=7, Rpa135-HTP *rpa12ΔC* n=4) or mean (Rpa135-HTP *nsi1ΔC* n=2, Rpa135-HTP *rpa12ΔC nsi1ΔC* n=2).

A: Depletion of Rnt1-AID-FLAG following indole acetic acid (IAA) addition to medium.

B: Distribution of RNAPI (Rpa135-HTP) CRAC 3’ ends of reads along the rDNA in the WT

C: Distribution of RNAPI (Rpa135-HTP) CRAC 3’ ends of reads along the rDNA in the *rpa12ΔC* strain
D: Distribution of RNAPI (Rpa135-HTP) downstream from the major T1 termination sites, comparing WT and *rpa12ΔC* strains. Differences are highlighted in color
E: Distribution of RNAPI Rpa135-HTP CRAC reads around the 3’ end of the 35S pre-rRNA.
F: RNAPI (Rpa135-HTP) CRAC signal is increased at the T2 site in the *rpa12ΔC* strain
G: Limited effect of *nsi1Δ* on RNAPI transcriptional read-through detected with Rpa135-HTP CRAC in the WT
H: Limited effect of *nsi1Δ* on RNAPI (Rpa135-HTP) transcriptional read-through in the *rpa12ΔC* background
I: Effect of *rpa12ΔC* on RNAPI (Rpa135-HTP) transcriptional read-trough in the *nsi1Δ* background

Figure S3

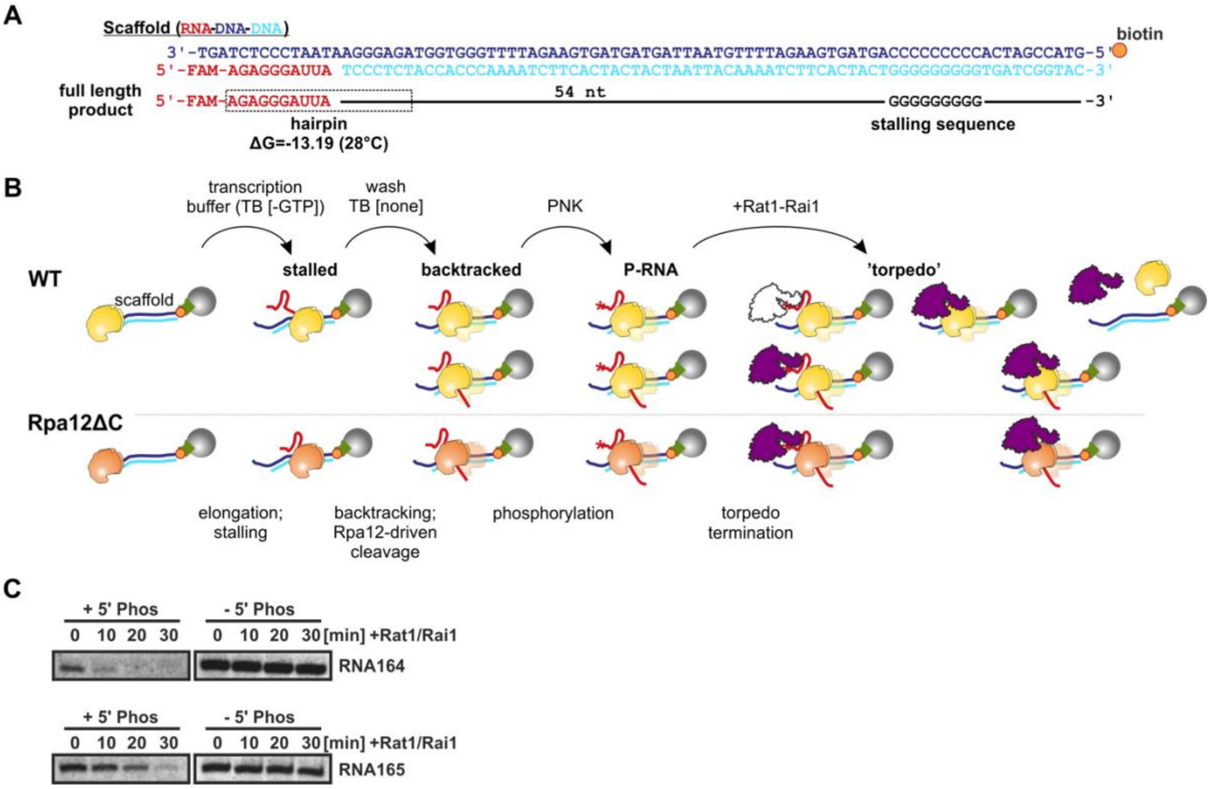

Figure S3. Schematic of *in vitro* termination assay (related to Figure 3).
A: Sequence of RNA-DNA-DNA scaffold immobilized via biotin-streptavidin interaction.
B: Schematic of the experiment.
C: Degradation of RNA oligonucleotides over a time course following addition of the purified Rat1-Rai1 complex, showing substantial protection in the absence of the 5’ monophosphate. Buffer and conditions as for termination assay (Fig. 3), but without addition of NTPs.

Figure S4

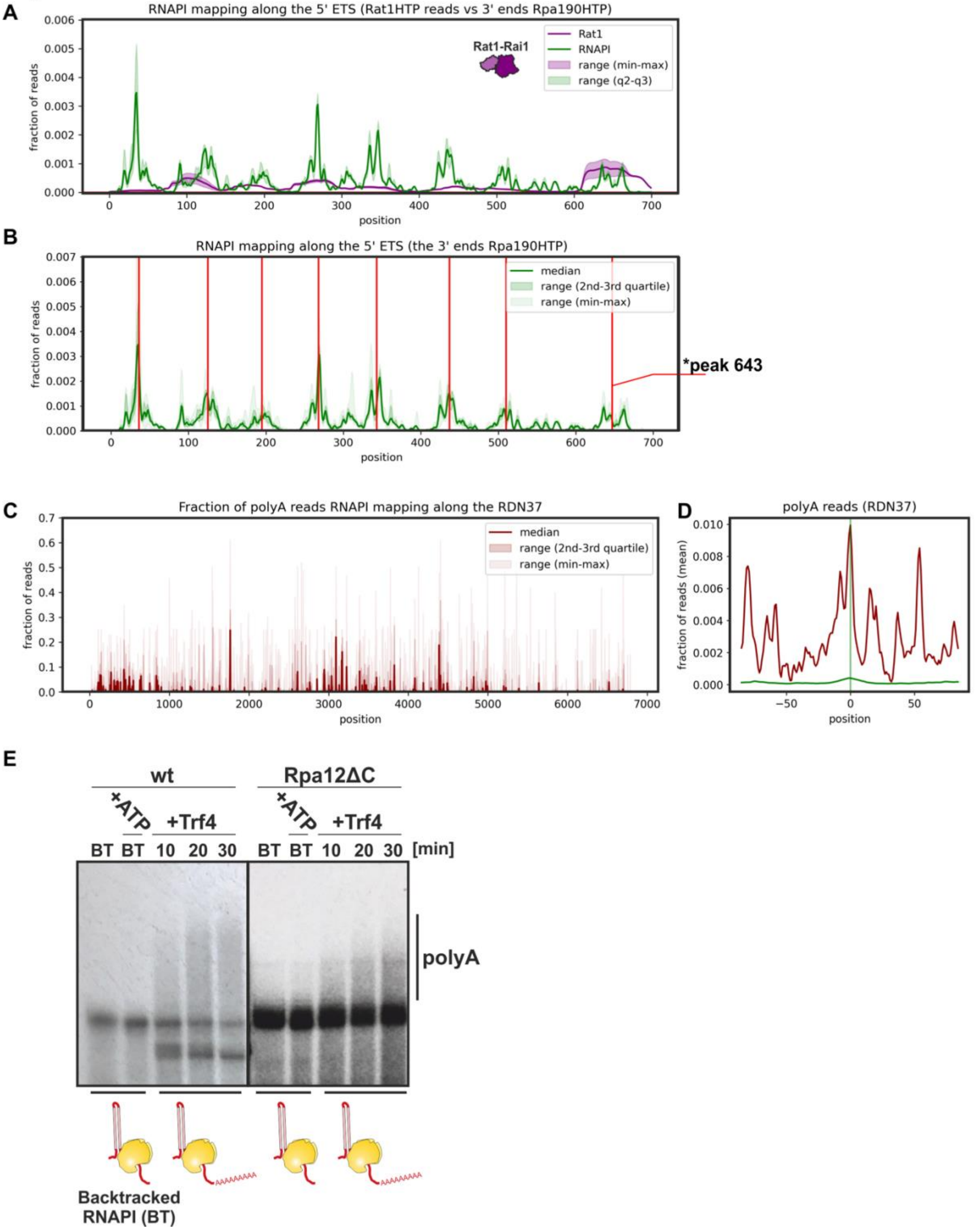

Figure S4. Rat1 and poly(A) tails are found at sites of slowed RNAPI elongation (related to Figure 4).
A: Rat1 total CRAC reads superimposed with the 3’ ends of RNAPI CRAC reads. Data are represented as mean (n=2).
B: Peaks found with peak-calling algorithm that were used to generate peak metaplot. * Note peak 643 was excluded from meta-analysis to separate Rat1 surveillance from canonical degradation of the 5’ ETS during pre-rRNA processing.
C: Fraction of poly(A) reads mapping along the rDNA (*RDN37*). Data are represented as mean (n=6).

D: RNAPI peak metaplot for *RDN37*, comparing the 3' ends of total Rpa190 reads (green) with poly(A) reads (red). As expected, the Rpa190 distribution matches the metapeak plot giving a low signal.

E: Trf4 oligo-adenylates the 3' end of backtracked, nascent RNA *in vitro* extruded from RNAPI. Comparison of adenylation on nascent transcripts associated with WT RNAPI (left) or RNAPI incorporating catalytically inactive Rpa12ΔC (right).

Figure S5

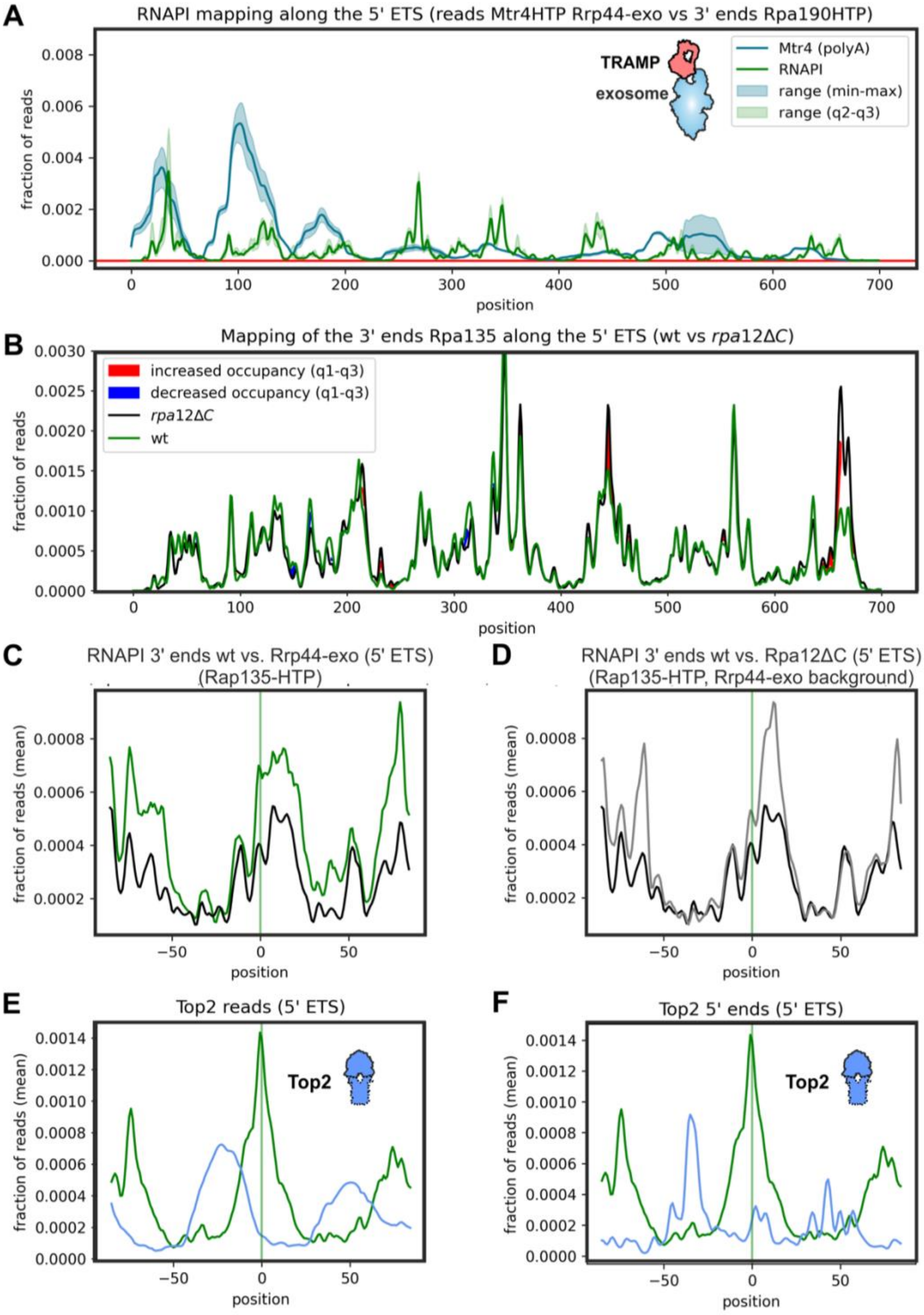

Figure S5. RNAPI and Top2 distributions along the 5’ ETS (related to Figure 5).
A: Mtr4 (Rrp44-exo) total CRAC reads (blue) superimposed over Rpa190 3’ end reads (green)s. Data are represented as mean (n=2).
B: Distribution of RNAPI (Rpa135-HTP) over the 5’ ETS comparing WT and *rpa12∆C* strains. Differences are highlighted in color. Data are represented as median (Rpa135-HTP n=7, Rpa135-HTP *rpa12∆C* n=4).
C: RNAPI (Rpa135-HTP) peak metaplot for the 5’ ETS, comparing WT (black) with *rrp44-exo (green)* strains
D: RNAPI (Rpa190-HTP) CRAC peak metaplot for the 5’ ETS, comparing WT with *rrp44-exo* (black) or *rpa12∆C* (gray) strains

E-F: Distribution of Top1-HTP CRAC (blue) over the 5’ ETS versus Rpa190 3’ ends (green) in a Rpa190 peak metaprofile: (E) total Top2 reads; (F) 5’ ends of Top2 reads

Figure S6

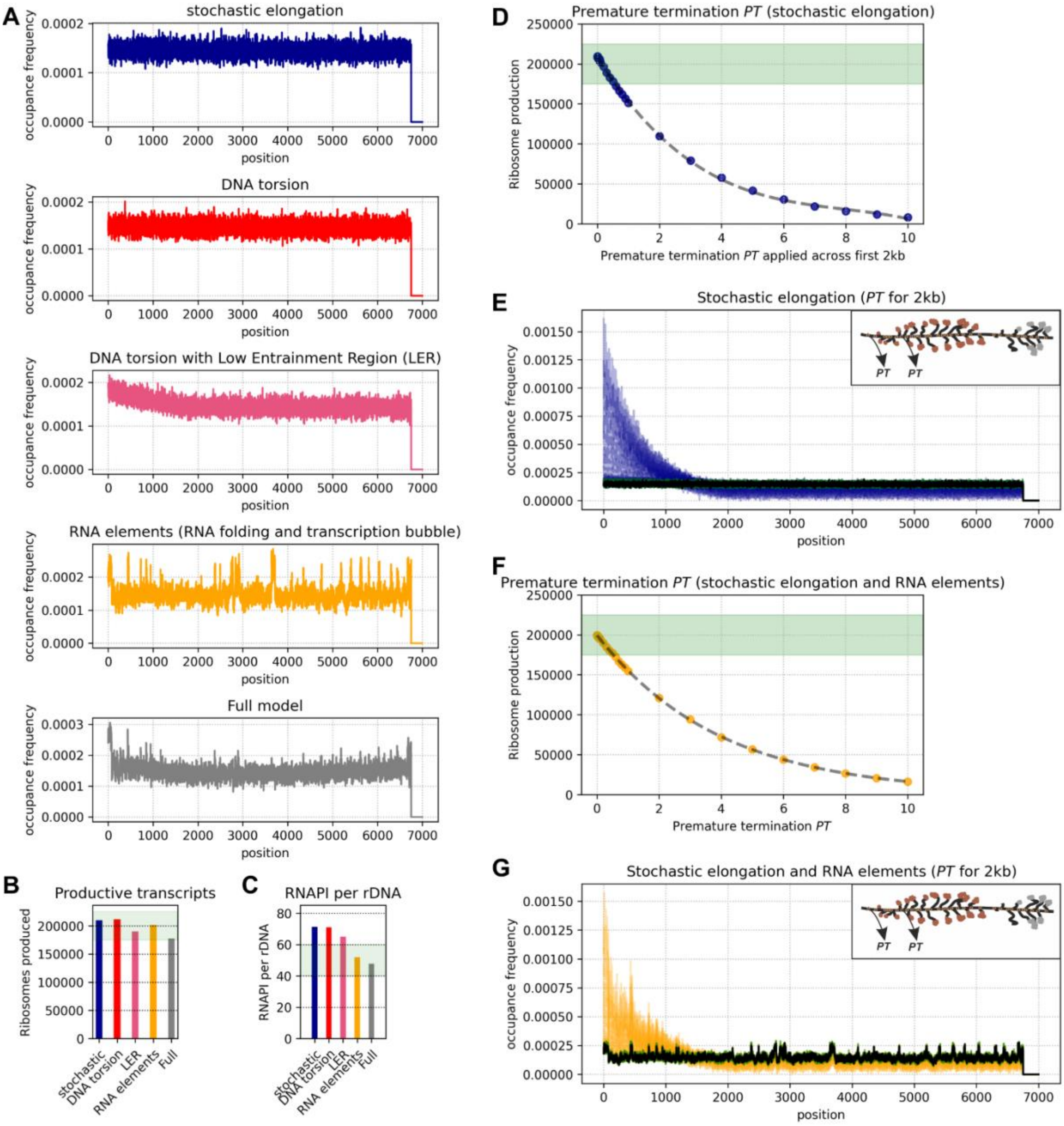

Figure S6. Development of premature termination P_{PT} function (related to Figure 6)
A: Modeled RNAPI occupancy along the transcription unit using a model of stochastic initiation and discrete, stochastic elongation. The average RNAPI occupancy for 16 simulations is presented. Each simulation was run for 6,000 sec and 200 time points were collected.
B: Average number of productive transcription events for each type of simulation (corresponding to panel A).
C: The average number of RNAP molecules per transcription unit (corresponding to panel A).
D: P_{PT} effect on ribosome production in model including stochastic elongation only. P_{PT} was applied across the first 2kb of the transcription unit. Note that because P_{PT} is applied only for this part of the transcription unit the values of P_{PT} are significantly higher than for panel Fig 6A.
E: RNAPI occupancy profiles corresponding to panel D. Black – no premature termination P_{PT}, green P_{PT} where ribosome synthesis is effective, blue – ribosome synthesis is decreased or insufficient.
F: P_{PT} effect on ribosome production in model including stochastic elongation and RNA elements. Note that because P_{PT} is applied only for the part of the transcription unit the values of P_{PT} are significantly higher than for panel Fig. 6A.

G: RNAPI occupancy profiles corresponding to panel E. Black – no premature termination P_{PT}, green P_{PT} where ribosome synthesis is effective, orange – ribosome synthesis is decreased or insufficient.

Table S1

ID	Name	loc	sequence
PCR062	RPA190-HTP tagging	fwd	ATGTTGGTACGGGTTCATTTGATGTGTTAGCAAAGGTT<br>CCAAATGCGGCTGAGCACCATCACCATCACC
PCR063	RPA190-HTP tagging	rev	ATGATGCACTTTATAATACCCTAGCTCTCTTAATTTACA<br>TAACACACGCGTACGACTCACTATAGGG
PCR064	Rpa190 check	fwd	TTTATCGACGTTGATGGTATTA
PCR065	Rpa190 check	rev	GCATTGACAGGCTCCATAA
PCR073	Rpa135-HTP tagging	fwd	CTGAGCTATCCGCAATGGGTATAAGATTGCGTTATAATG<br>TAGAGCCCAAAGAGCACCATCACCATCACC
PCR074	Rpa135-HTP tagging	rev	ACAATTGACCAAGCCTTCATTTACCATTCTATATCAATTTG<br>GAAAGAAGGGTATACGACTCACTATAGGG
PCR075	Rpa135 check	fwd	ATGTCGCGAGTGTGGTTCTATTTT
PCR076	Rpa135 check	rev	AAGCCTGCACCTCTTGCAGTAGAA
EP98	Nsi1 F1 deletion	fwd	CAAATTTTGTGCATAGAGCAAGCAGCCGTTTCTTGTCT<br>GCGTCAAGAAGAAAGATAAAGGTAGACGGATCCCCGG<br>GTTAATTAA
EP99	Nsi1 R1 deletion	rev	TTTAAAAATCAGTAAATATGCTTTTATCTATTGGGTCTGT<br>ATATGTTTGGGAAAGTAACCCTTCGAATTCGAGCTCGT<br>TTAAAC
EP100	Nsi1 test	fwd	GAGCTTTCCAAATGCGATA
JH1584	MX4-6 rev		TGCAGCGAGGAGCCGTAAT
EP97	Rai1 TAPfwd	fwd	TCTCGGAATAACAAGCAAAGTCGGTATGACAATTCAAG<br>AGCAAATAGGCGTTCCATGGAAAAGAGAAG
EP96	Rai1Tap rev	rev	TTTATAAATTTGCGAAAACCTAAATTTACCATAAAATGC<br>GCACGAGTAGTTTACGACTCACTATAGGG
PCR062	Rpa190HIS	fw	ATGTTGGTACGGGTTCATTTGATGTGTTAGCAAAGGTT<br>CCAAATGCGGCTGAGCACCATCACCATCACC
PCR063	Rpa190TAP	rev	ATGATGCACTTTATAATACCCTAGCTCTCTTAATTTACA<br>TAACACACGCGTACGACTCACTATAGGG
PCR255	pMet-AID-6Flag-Rnt1	fwd	TGCGCATATAGAAGAGAGCAAAACTGTCCTATTTACAA<br>GCTTTTCAAAACAGACATGGAGGCCCAGAATA
PCR256	pMet-AID-6Flag-Rnt1	rev	GTTTATTATCATTCTGGGTTTTCTTTTTACCTGCTACTTT<br>TGAGCCCATATCTGGCGCGCCACATCTAGA
PCR801	RRP44_D551 CRISPR plasmid	fwd	GATCTCAATATCAACACATCCTGGGTTTTAGAGCTAG
PCR802	RRP44_D551 CRISPR plasmid	rev	CTAGCTCTAAAACCCAGGATGTGTTGATATTGA
PCR805	RRP44_D551 HDR	fwd	Gacaaaaagaaaggatctcagagataaacttatatgtagtatcgatcctcca<br>ggCtgCgtCgatattAAT
PCR806	RRP44_D551 HDR	rev	Acaccaacttcccaattaccgtttggaagctttttcgcatgtagggcat<br>cATTaatatcGacGcaGcctg

Table S1. Oligonucleotides used for strain construction.

Table S2

ID	oligo	sequence
oTWT039	5'FAM	AGGCCGAAA
oTWT114		/56-FAM/rArGrArGrGrGrArUrUrA
oTWT150bio		/5Biosg/ATACTTACAGCGTACCGATCACCCCCCCCCAGTAGTGA AGATTTTGTAATTAGTAGTAGTGAAGATTTTGGGTGGTAGAGGG AATAATCCCTCTAGT
oTWT151		TCCCTCTACCACCCAAAATCTTCACTACTACTAATTACAAAATCT TCACTACTGGGGGGGGGTGATCGGTAC
oTWT164		rUrCrUrArCrGrUrArUrArArGrArGrGrGrArUrUrA
oTWT165		rUrArUrArUrCrUrUrGrUrCrArArUrCrArUrArCrCrArGrArGrGrGrArUrUrA

Table S2. Oligonucleotides used for *in vitro* assays.
