# Supplementary material for: Multiple mechanisms of termination modulate the dynamics of RNAPI transcription
Source: Cell Rep. Author manuscript; Available in PMC 2026 Feb 3. (PMC7618708; doi:10.1016/j.celrep.2025.115325)
Supplement: Supplementary Material [file EMS212091-supplement-Supplementary_Material.zip › 1-s2.0-S2211124725000968-mmc2.pdf]

Cell Reports

Article

# Multiple mechanisms of termination modulate the dynamics of RNAPI transcription

## Graphical abstract

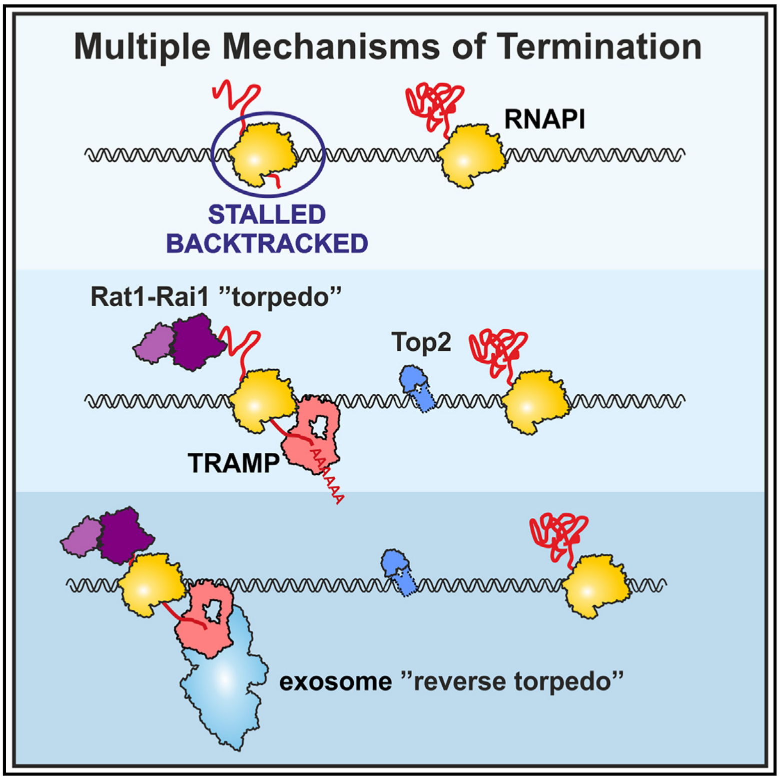


## Authors

Elisabeth Petfalski, Marie-Luise Winz, Katarzyna Grelewska-Nowotko, Tomasz W. Turowski, David Tollervey

## Correspondence

tomasz.turowski@ibb.waw.pl (T.W.T.), d.tollervey@ed.ac.uk (D.T.)

## In brief

Petfalski et al. reveal complexities in RNAPI termination. Torsion release, through nascent pre-rRNA cleavage by Rnt1, favors RNAPI pausing and torpedo termination by the exonuclease Rat1, aided by Nsi1 roadblocks and RNAPI-mediated endonuclease cleavage. Termination of prematurely stalled RNAPI is facilitated by TRAMP-mediated nascent transcript polyadenylation and a reverse torpedo mechanism.

## Highlights

- Nascent pre-rRNA 3′ cleavage promotes RNAPI deceleration and termination
- RNAPI undergoes early start-site proximal termination at sites of polymerase pausing
- Biophysical modeling indicates ∼10% early termination of RNAPI
- Model supports roles of additional rDNA repeats in buffering pre-rRNA transcription

Petfalski et al., 2025, Cell Reports *44*, 115325
March 25, 2025 © 2025 The Authors. Published by Elsevier Inc.
https://doi.org/10.1016/j.celrep.2025.115325

CellPress

Cell Reports

CellPress
OPEN ACCESS

Article

# Multiple mechanisms of termination modulate the dynamics of RNAPI transcription

Elisabeth Petfalski,[1] Marie-Luise Winz,[1,4] Katarzyna Grelewska-Nowotko,[2] Tomasz W. Turowski,[2,3,*] and David Tollervey[1,3,5,*]
[1]Centre for Cell Biology, School of Biological Sciences, The University of Edinburgh, Michael Swann Building, Edinburgh EH9 3BF, UK
[2]Institute of Biochemistry and Biophysics PAS, Pawińskiego 5A, 02-106 Warszawa, Poland
[3]These authors contributed equally
[4]Present address: Pharmaceutical and Medicinal Chemistry, Institute of Pharmaceutical and Biomedical Sciences (IPBS), Johannes Gutenberg University Mainz, Staudingerweg 5, 55128 Mainz, Germany
[5]Lead contact
*Correspondence: tomasz.turowski@ibb.waw.pl (T.W.T.), d.tollervey@ed.ac.uk (D.T.)
https://doi.org/10.1016/j.celrep.2025.115325

## SUMMARY

Transcription elongation is stochastic, driven by a Brownian ratchet, making it subject to changes in velocity. On the rDNA, multiple polymerases are linked by "torsional entrainment" generated by DNA rotation. We report that release of entrainment by co-transcriptional 3′ end cleavage, is permissive for relative movement between polymerases, promoting pausing and backtracking. Subsequent termination (polymerase release) is facilitated by the 5′ exonuclease Rat1 (Xrn2) and backtracked transcript cleavage by the RNA polymerase I (RNAPI) subunit Rpa12. These activities are reproduced *in vitro*. Short nascent transcripts close to the transcriptional start site, combined with nascent transcript folding energy, similarly facilitate RNAPI pausing. Nascent, backtracked transcripts at pause sites are terminated by forward and reverse "torpedoes": Rat1 and the exosome cofactor Trf4/5-Air1/2-Mtr4 polyadenylation (TRAMP), respectively. Topoisomerase 2 localizes adjacent to RNAPI pause sites, potentially allowing continued elongation by downstream polymerases. Mathematical modeling supported substantial premature termination. These basic insights into transcription *in vivo* will be relevant to many systems.

## INTRODUCTION

The pre-ribosomal RNA (pre-rRNA; 7 kb in yeast, 10 kb in humans) encodes the 18S, 5.8S, and 25S/28S rRNAs flanked by the 5′ and 3′ external transcribed spacers (ETSs) and separated by internal transcribed spacer 1 (ITS1) and ITS2 (Figures 1A and S1A). Ribosome synthesis initiates co-transcriptionally with assembly of the nascent pre-ribosomes during pre-rRNA transcription (Figure 1B). In actively growing yeast, each transcribed ribosomal DNA (rDNA) is typically associated with around 50 RNA polymerase I (RNAPI) molecules (Figure 1B). Transcription elongation is composed of many successive cycles of nucleotide addition, in which the translocation step is based on Brownian motion without input of external energy. Dependence on this "Brownian ratchet," rather than an energy-driven processive mechanism, makes elongation prone to frequent backtracking and potentially sensitive to inhibition or acceleration by quite modest forces.[1–3] In the case of RNAPII, pausing and backtracking is common and a major factor in overall gene expression.[4–6]

We previously determined the distribution of transcribing RNAPI across the rDNA, comparing the densities revealed by analyses of "Miller" chromatin spreads, RNAPI chromatin immunoprecipitation, and the UV-crosslinking and analysis of cDNAs (CRAC) technique.[7,11] Two key features emerged: an apparent excess of RNAPI over the 5′ region of the pre-rRNA and very uneven polymerase distribution with marked troughs and peaks, particularly over the 5′ region (Figure 1C). The strikingly uneven distribution of transcribing RNAPI (Figure 1C) largely reflected positive effects of nascent RNA folding in promoting rapid transcription elongation, with peaks of polymerase density (slow elongation) correlating with weak folding in a 65 nt window of extruded RNA behind the polymerase.[7,12] Additionally, one complete rotation of the DNA is required for each ∼10.3 nt synthesized. At the reported elongation rate (∼40 nt $s^{-1}$),[13] this will occur at around 230 rpm, with ∼680 complete rotations required for each pre-rRNA molecule transcribed. The large (megadalton) nascent pre-ribosomes are expected to effectively block rotation by the polymerase complex, so the polymerase array will collectively spin the rDNA. Moreover, any relative movement between polymerases on the same rDNA is blocked by the buildup of torsion between each of them. This "torsional entrainment" has less effect over the 5′ region of the rDNA, since the short nascent transcripts are predicted to allow greater freedom for rotation by the polymerases, facilitating relative movement along the rDNA (Figure 1D).[7] This is permissive for changes in the relative positions of the polymerase, allowing the large peaks and troughs in RNAPI density seen over the initiation-proximal region (Figure 1C). Consistent with strong torsion, RNAPI transcription also requires


© 2025 The Authors. Published by Elsevier Inc.
This is an open access article under the CC BY license (http://creativecommons.org/licenses/by/4.0/).

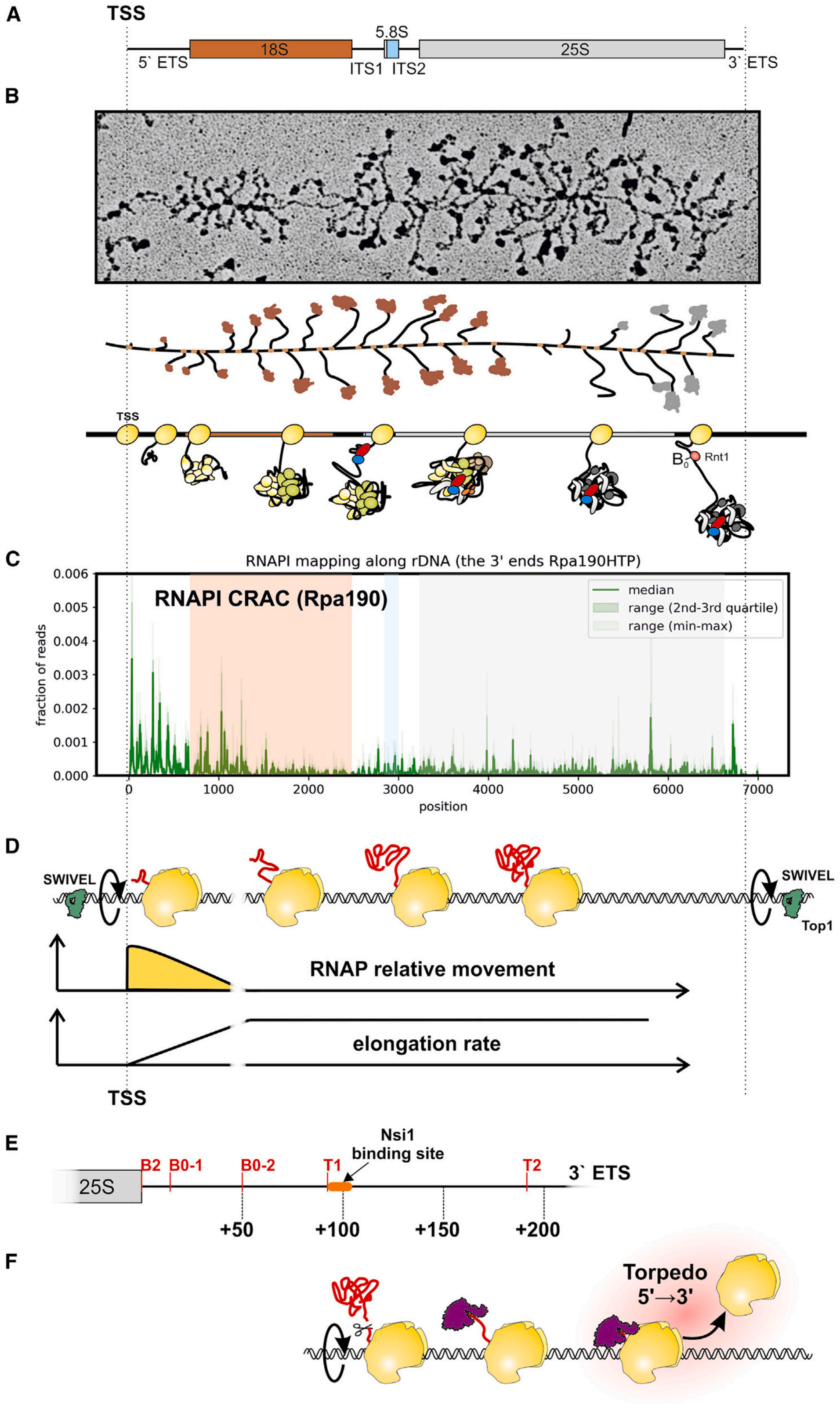


(legend on next page)

topoisomerase activity, with elongation blocked in the 5′ region of the rDNA by combined depletion of topoisomerase 1 (Top1) and Top2.[11,8] Top1 binds the rDNA both 5′ and 3′ of the transcription unit, recruited by the DNA binding protein Fob1.[14–16] We postulate that Top1 at these sites acts as a swivel, allowing the entire rDNA unit to spin (Figure 1D).

Previous analyses of yeast RNAPI transcription *in vitro* and *in vivo* in yeast showed that ~90% of transcripts terminate at site T1, a T-rich element located ~93 nt downstream of the 3′ end of the 25S sequence (site B2) (Figure 1E).[17–20] Termination requires co-transcriptional cleavage by Rnt1, an RNase III family member, across a stem-loop structure within the 3′ ETS at positions +14/15 and +49/50 (sites B0-1 and B0-2) relative to the 3′ end of the 25S rRNA sequence (Figure 1E).[21–23] B0 cleavage allows entry of the processive nuclear 5′-3′ exonuclease Rat1 (Xrn2 in humans), which degrades the nascent transcript and provokes "torpedo" termination (Figure 1F). Binding by NTS1 silencing protein 1 (Nsi1) at a site 12–20 nt downstream of T1 also contributes to efficient termination.[17,24–26]

Deletion of the *RPA12* gene, which encodes a small subunit of RNAPI, also leads to increased readthrough of T1.[18] Rpa12 is implicated in RNAPI transcription initiation and elongation and in the cleavage of the nascent transcript when this is in a backtracked position.[27–29] Deletion of only the C-terminal domain of Rpa12 separates these functions with a loss of cleavage activity but minimal effects on RNAPI core activity.[30–32]

Here, we report that termination is favored by release of torsional entrainment through 3′ cleavage and by polymerase pausing early in the transcription unit.

## RESULTS

### RNAPI distribution indicates very rapid transcription termination following 3′ cleavage

During pre-rRNA transcription, multiple polymerases on each active rDNA form a "convoy," moving together. Any change in the elongation rate or position of a single RNAPI molecule within the convoy requires it to rotate relative to the rest of the convoy. Over much of the rDNA, RNAPI is linked to very large pre-ribosomal complexes, including kilobases of RNA, greatly constraining such rotation. However, short nascent transcripts present on RNAPI in the 5′ ETS close to the transcription start site (TSS), are expected to allow greater freedom for rotation around the rDNA and relative movement (Figure 1D).[7] This is permissive for greater variability in elongation rates within this region, as shown by heterogeneous RNAPI occupancy (Figure 1C).

RNAPI can be mapped with nucleotide resolution at the 3′ ends of nascent transcripts, using CRAC in strains expressing Rpa190 (catalytic subunit) or Rpa135 as fusions with a C-terminal $His_6$-TEV-protein A (HTP) affinity purification tag.[7] This revealed a clear peak of polymerase density in the 3′ ETS (Figure 2A, green line). Previous analyses revealed a strong correlation between the rate of transcription elongation and strength of RNA folding within the extruded nascent transcript.[7,12] We proposed that formation of stable stem structures promotes elongation and resists backtracking by RNAPI. This correlation was particularly strong over the 5′ ETS region but was clearly visible across the pre-rRNA. Co-transcriptional cleavage of the nascent transcript can occur within the 25S rRNA region,[13] potentially also favoring slowed elongation here.

The 3′ ETS region was notable, since there is strong folding of the nascent transcript (low $\Delta G$; Figure 2A, yellow line) flanking a site of high RNAPI occupancy and, therefore, a low elongation rate (Figure 2A, green line). A stable stem is certainly present at this position *in vitro*, since it forms the extended, double-stranded structure essential for Rnt1 cleavage.[21–23] Note that the yellow line shows the folding energy for the 65 nt of extruded RNA located 5′ of each position, not the folding energy at the site.

Within the 3′-ETS, the peak of polymerase density is located between the B0 cleavage site and terminator T1. To better understand this location, we mapped the 5′ ends of CRAC reads (Figures 2B, black line, S1B, and S1C). This revealed a very strong peak at the 3′ Rnt1 cleavage site (B0-2, black dot). Strikingly, reads with 5′ ends at site B0-2 had a median length of 25 nt (Figure 2C). This would be consistent with very rapid co-transcriptional cleavage of the nascent pre-rRNA by Rnt1 (Figure 2C). We cannot formally exclude the possibility that some Rnt1 cleavage occurs post lysis, but the low temperature (ice or 4°C) and absence of $Mg^{2+}$ in the lysis buffer make this unlikely. Notably, the major RNAPI-associated peak of B0-2 cleaved RNA did not extend to the reported termination site at T1 (Figure 2B, red line), indicating that the 3′-end is a site of transcriptional pausing or backtracking. Nascent transcript cleavage has been reported at site T1,[33] but this was not evident in our data, as no clear 5′ end peak was observed at this position.

Transcription termination has been linked to pausing and backtracking of the polymerase.[4,6,34] This should be suppressed by torsional entrainment, since each polymerase complex is "pushed" and "pulled" by all others on the transcription unit (typically around 50 for the rDNA)[7,35–37] and anchored in the nucleolar condensate by the nascent pre-ribosomes. In the 3′ ETS region, the pre-rRNA undergoes co-transcriptional cleavage by Rnt1.

**Figure 1. RNAPI convoys are torsionally entrained on spinning DNA**

(A) Schematic of the 35S pre-rRNA.

(B) Image and schematics of the rDNA with RNAPI and nascent pre-ribosomes.

(C) Distribution of RNAPI CRAC reads along the rDNA, highlighting the peak at the 3′ end of the 35S pre-rRNA. Rpa190-HTP CRAC data re-processed from Turowski et al.[7] Data are represented as median, 25%–75% quantile range, and minimum-maximum range ($n$ = 6).

(D) Cartoon of the modeled RNAPI elongation rate along the rDNA. Top: multiple RNAPI molecules are torsionally entrained on the DNA, which is spinning rapidly (~230 rpm) during transcription elongation. Topoisomerase 1 (Top1) sites flanking the transcription unit release DNA torsion by introducing single-strand nicks in DNA.[8,9,10] Center: cartoon of implementation of the low-entrainment region (LER): initial ability of RNAP to spin at the beginning of the transcription unit. Bottom: a consequence of the LER is a progressive increase of RNAP velocity at the beginning of the transcription unit.

(E) Schematic showing processing sites within the 3′ ETS: the B2 site at the very 3′ end of 25S rRNA, two B0 sites that are cleaved together across a stem structure by the RNase III homolog Rnt1, termination sites T1 and T2, and the binding site of the roadblock terminator Nsi1.

(F) Cartoon illustrating Rat1-Rai1 torpedo termination of RNAPI transcription.

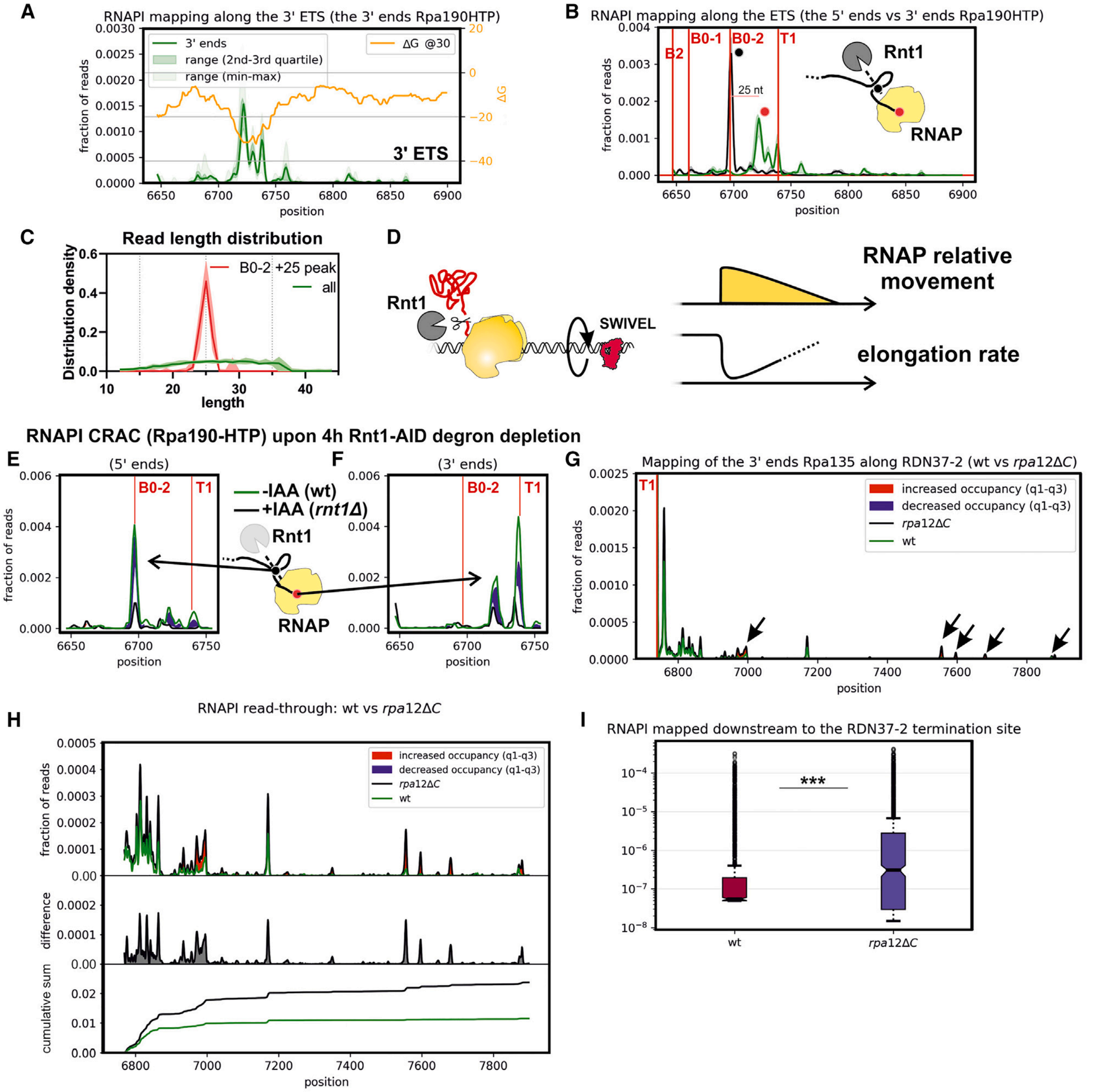


**Figure 2. RNAPI transcription pausing in the 3′ ETS**

(A) Green line, distribution of RNAPI CRAC reads around the 3′ end of the 35S pre-rRNA; yellow line, folding energy in a rolling window of 65 nt, offset by 15 nt (the region within the polymerase) behind the polymerase at each nucleotide position. Data are represented as median ($n$ = 6).

(B) Distribution of RNAPI CRAC reads around the 3′ end of the 35S pre-rRNA. Rnt1 processing sites (B0-1 and B0-2) and the reported major terminator (T1) are indicated. Black line, distribution of 5′ ends of RNAPI CRAC reads around the 3′ end of the 35S pre-rRNA. The major peak corresponds precisely to the 3′ B0 cleavage site. Green line, distribution of RNAPI CRAC read 3′ ends. The peaks of the 5′ and 3′ end distributions are positioned 25 nt apart. A cartoon shows the two B0 sites that are cleaved together across a stem structure by the RNase III homolog Rnt1. Peaks corresponding to the major 5′ (black) and 3′ (green) peaks are labeled on the plot and in the cartoon with round markers. Data are represented as median ($n$ = 6).

(C) Read length distribution plot for all reads mapped to *RDN37-2* (all) and reads mapped at the major peak within the 3′ ETS (B0-2 + 25 peak), marked with green in (B).

(D) Cartoon showing a decreased RNAPI elongation rate as a result of Rnt1 cleavage. RNAPI is released from torsional entrainment on the DNA by Rnt1 cleavage (top). This facilitates rotation of RNAPI together with the DNA (center) and allows a decreased elongation rate (bottom).

(E) Mapping 5′ ends of RNAPI (Rpa190-HTP) CRAC data reveals that AID-Rnt1 depletion is associated with loss of B0-2 processing ends within the nascent RNA. This confirms that RNAPI-associated reads are co-transcriptionally cleaved by Rnt1. Data are represented as median ($n$ = 3).

(legend continued on next page)

We postulated that release from the pre-60S would increase the capacity of RNAPI to spin together with the rDNA. This could facilitate transcriptional deceleration and favor pausing/backtracking, contributing to termination (Figure 2D).

To test the requirement for Rnt1 cleavage in RNAPI pausing, we constructed a strain in which Rnt1 is fused to the auxin-induced degron (AID) and a FLAG tag.[38] In this strain, AID-FLAG-Rnt1 is the only source of Rnt1, and no clear growth defect was seen under permissive conditions. Following addition of the auxin analog indole acetic acid (IAA), AID-FLAG-Rnt1 was substantially depleted at 2 h and almost undetectable after 4 h (Figure S2A). The Rpa190-HTP CRAC analysis was repeated in the AID-FLAG-Rnt1 strain following IAA addition for 4 h (Figures 2E and 2F). Rnt1 depletion greatly reduced the formation of the major 5′ end of CRAC reads at site B0 (an Rnt1 cleavage site), as expected (Figure 2E). It also strongly suppressed recovery of transcripts with 3′ ends corresponding to the peak upstream of T1 (Figure 2F).

Deletion of the gene encoding Rpa12 was shown to increase transcription readthrough of site T1 in Miller spreads[18] but also greatly reduces overall pre-rRNA synthesis. Rpa12ΔC, deleted for the C-terminal domain, lacks nascent transcript cleavage activity but retains Rpa12 function in RNAPI elongation.[30–32] We therefore assessed the effects of Rpa12ΔC truncation on RNAPI occupancy across the rDNA. In these analyses, HTP-tagged Rpa135[7] was used to map RNAPI (Figures S2B–S2F). Strains with *rpa12ΔC* showed an elevated CRAC signal for RNAPI across the 3′-ETS, site T1, and intergenic spacer region 1 (Figures 2G, 2H, and S2D; quantified in Figures 2I and S2F). We conclude that cleavage of the backtracked nascent transcript within RNAPI contributes to efficient termination. However, the major RNAPI peak of occupancy remained downstream of the Rnt1 cleavage sites.

The DNA-binding protein Nsi1 binds a region 12–20 nt 3′ of site T1[17,24–26] and mediates "roadblock" termination. To assess the extent to which the 3′ ETS peaks of RNAPI occupancy reflect pausing enforced by Nsi1, RNAPI CRAC was repeated in an *nsi1Δ* strain and in an *nsi1Δ, rpa12ΔC* double mutant (Figure S2). Relative to the wild type, RNAPI occupancy downstream of site T1 was elevated in the *nsi1Δ* strain and further increased in the *nsi1Δ, rpa12ΔC* double mutant (Figures S2G–S2I). However, the strong peak of RNAPI occupancy upstream of site T1 remained in all strains.

We conclude that both nascent transcript cleavage by Rpa12 and transcription pausing promoted by Nsi1 contribute to efficient termination in addition to degradation by Rat1. The peak of RNAPI density downstream of the Rnt1 co-transcriptional cleavage site was not strongly dependent on the presence of Nsi1, consistent with a large contribution from loss of torsional entrainment following pre-ribosome release.

### Reconstitution of transcription termination *in vitro*

To better characterize the role of nascent RNA degradation in triggering RNAPI termination, we developed an *in vitro* system (outlined in Figure 3A and shown in more detail in Figure S3). We previously reported a system for *in vitro* transcription and backtracking by RNAPI that was analyzed by following the RNA products of the reactions.[7] We designed a strong structural element in the nascent transcript that allows to block backtracking of the polymerase and, subsequently, exchange reaction buffer. To assess transcription termination, we modified this system to measure release of the polymerase from the DNA template in addition to the RNA product. To achieve this, we immobilized the double-stranded template DNA on streptavidin beads via the template strand, allowing termination to be monitored by release of the transcribing polymerase into the supernatant. The elongating transcription complex was stalled and allowed to backtrack, followed by degradation of the nascent transcript. The 5′ exonuclease Rat1 normally functions in a stoichiometric complex with the pyrophosphatase Rai1,[39,40] and we therefore purified the Rat1-Rai1 complex via TAP-tagged Rat1. For *in vitro* transcription, we generated a construct with a G9 element (Figure S3A) and induced stalling by transcription in the absence of guanosine triphosphate. Rat1 is most active on substrates with a 5′ monophosphate, so this was added to the transcription primer by polynucleotide kinase treatment (Figures 3A and S3B). To confirm that nascent transcript degradation is indeed mediated by Rat1, we compared RNA degradation under assay conditions with substrates with and without a 5′ monophosphate (Figure S3C). The absence of a phosphate conferred almost complete protection, strongly indicating that Rat1 is the active nuclease.

Recovery of RNAPI in the streptavidin/DNA-bound fraction (B; not terminated) and supernatant fraction (S; terminated) was compared during a time course of Rat1/Rai1 treatment (Figure 3B; quantified in Figure 3C; see STAR Methods for details). This showed a significant increase in termination induced by transcript degradation. Rpa12 is an integral component of RNAPI. To assess the role of nascent cleavage in transcript termination *in vitro*, the assay was repeated using RNAPI purified from an *rpa12ΔC* strain. Using the Rpa12ΔC form of the polymerase, significant enhancement of termination was not seen following Rat1/Rai1 treatment (Figures 3B and 3C), consistent with our *in vivo* data (Figures 2E and 2F).

These results show that both Rat1 and Rpa12 activities are needed for efficient torpedo termination *in vitro* (Figure 3D). Rat1 shows highly processive 5′ exonuclease activity *in vitro*.[41]

(F) Comparison of RNAPI (Rpa190-HTP) 3′ mapping before and after AID-Rnt1 degron depletion. Rnt1 depletion leads to loss of the RNAPI peak between B0-2 and T1 sites. Solid lines indicate the median of −IAA (green) and +IAA (black). Blue filling indicates significant differences between datasets, which do not overlap with q2-q3 of each dataset. Data are represented as median ($n = 3$).

(G) RNAPI (Rpa135-HTP) CRAC shows increased readthrough in a *rpa12ΔC* strain in which endonuclease cleavage of backtracked transcripts is abrogated. Data are represented as median (WT, $n = 7$; *rpa12ΔC*, $n = 4$).

(H) *rpa12ΔC* leads to less efficient transcription termination of RNAPI. The plot compares WT and *rpa12ΔC* distribution (top) with differences extracted and plotted separately (center) and cumulative sum plots plotted (bottom). In *rpa12ΔC*, the signal is elevated downstream of termination site T1. Data are represented as median (WT, $n = 7$; *rpa12ΔC*, $n = 4$).

(I) Boxplot showing a fraction of reads downstream of the RDN37-2 termination site for WT and *rpa12ΔC* strains ($p < 0.001$, two-sided Wilcoxon test).

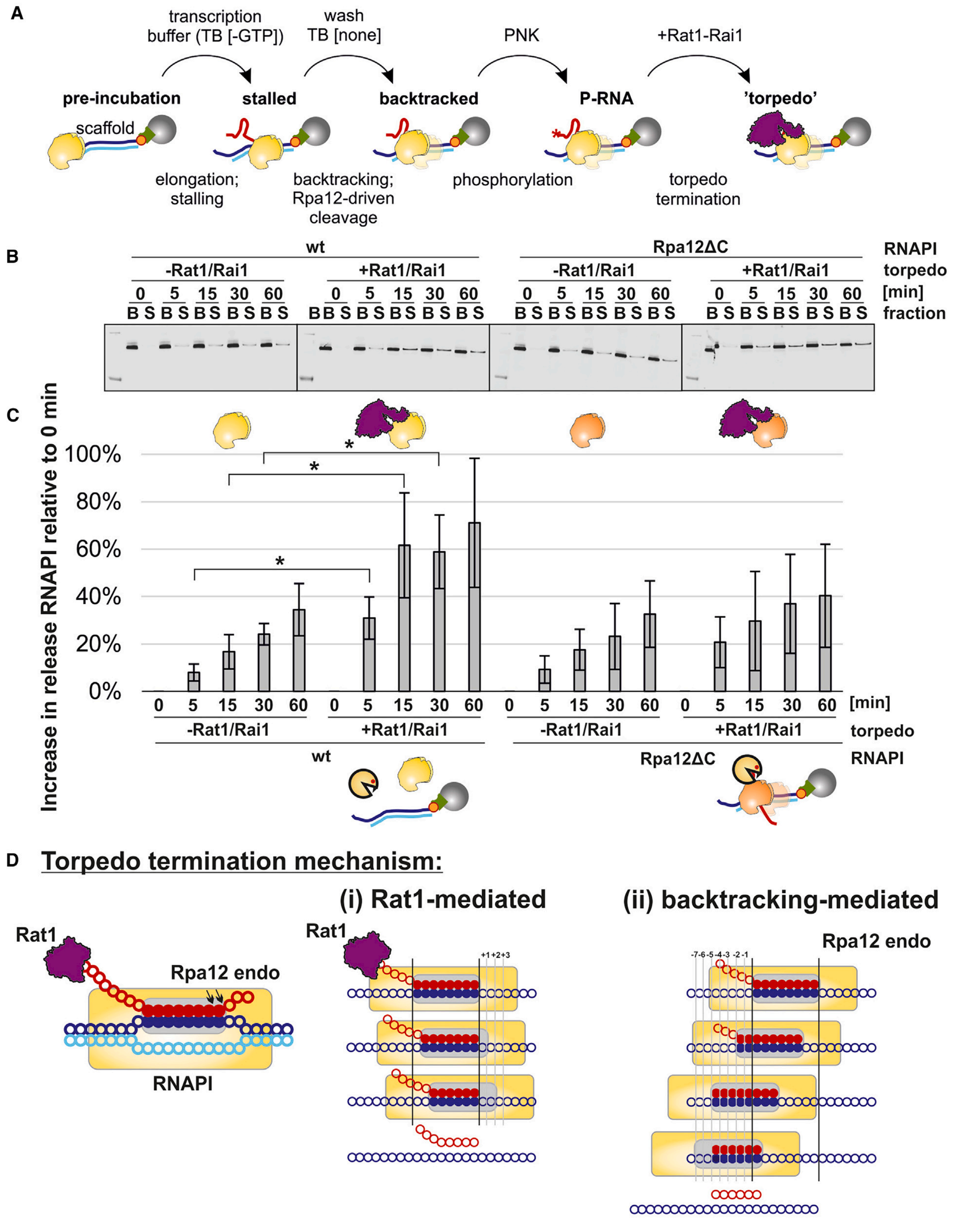


*(legend on next page)*

In principle, the RNAPI elongation complex could be pushed forward, without nucleotide incorporation, to the position where the transcription bubble becomes unstable and favors RNAPI dissociation (Figure 3D, i). However, this requires DNA strand separation without compensatory RNA-DNA base pairing, and it is unclear whether sufficient force can be generated by Rat1-Rai1. We propose an additional mechanism whereby the torpedo trims nascent RNA only to the boundary of the elongation complex, with further steps based on a backtracking-mediated mechanism until the transcription bubble becomes unstable (Figure 3D, ii). This mechanism would be favored by the elongation complex, which can "slide" backward on the DNA. There would, however, be competition between transcript elongation and endonuclease activity of the polymerase.

## Early termination by RNAPI

Termination in the 3′ ETS, the end of the transcription unit, appears to be associated with polymerase pausing and/or backtracking. Previous studies also indicated substantial peaks of RNAPI occupancy in the 5′ ETS, which we interpreted as indicating pause sites.[7,11,42] Our previous analyses indicated that the heterogeneity in elongation rates was linked to the reduced levels of torsional entrainment allowing the effects of nascent RNA folding to be strongly manifested. However, they did not exclude the possibility that some premature termination also occurs for RNAPI, as reported previously for RNAPII.[43,44]

Premature transcription termination is expected to be associated with degradation of the nascent transcript. During normal pre-rRNA processing, the excised 5′ A0 region of the 5′ ETS is degraded by the exosome together with the DExH RNA helicase Mtr4,[45,46] which is directly recruited by the pre-ribosome component Utp18.[47] RNA surveillance activities of the yeast nuclear exosome require the TRAMP (Trf4/5-Air1/2-Mtr4 polyadenylation) complex, which facilitates RNA degradation by addition of a single-stranded oligo(A) tail. However, TRAMP components other than Mtr4 are not required for pre-rRNA processing (5.8S trimming and 5′ ETS degradation). The 5′ exonuclease Rat1 is not a major factor for normal degradation of the 5′A0 region, probably reflecting a degree of protection conferred by the 5′ tri-phosphate on the primary transcript,[48] but degrades the excised A0-A1 region.[49]

Mapping the distribution of Rat1 across the 5′ ETS revealed a pattern of peaks (Figures 4A and S4A). Reads were mapped using the 5′ ends of the recovered sequences, since this expected to correspond to the location of Rat1. The strongest peak was at +610, corresponding to the 5′ end of the excised A0-A1 pre-rRNA region. Since yeast rDNA sequences are generally identical, metagene analyses cannot be applied. However, we have previously used a peak-calling algorithm to identify common features at different sites within the rDNA,[7] as outlined in Figure S4B. Using this approach, we identified and aligned peaks of occupancy for Rat1 (Figure 4B, purple line) and RNAPI (Figure 4B, green line) (excluding peak 643; Figure S4B). Rat1 occupancy showed a peak ∼40 nt upstream of the peak of RNAPI occupancy, mapped using the 3′ ends of the sequences. We interpret this as showing Rat1-Rai1 degradation of nascent transcripts associated with stalled/paused RNAPI or released by Rpa12 cleavage. We also evaluated the impact of the previously reported conditional *rat1-1* mutant[50] on RNAPI density within the 5′ ETS (Figure 4C). The *rat1-1* mutation resulted in a change in RNAPI peak distributions, observed as a more heterogeneous distribution of the 5′ ETS peaks, and additional accumulation downstream of peaks annotated in the wild type (WT) (Figure 4C). We interpret this as indicating pausing at additional sites in the rDNA in the *rat1-1* mutant. Overall, these results seem to be consistent with the occurrence of torpedo termination within the 5′ ETS.

## Adenylation of nascent transcripts

RNAPI peaks in the 5′ ETS correspond to sites of high occupancy, reflecting greatly slowed or paused elongation. We speculated that backtracking at these sites might expose the 3′ ends of nascent transcripts to the surveillance machinery. TRAMP-mediated surveillance tags substrate RNAs with oligo(A) tails, so we sought these in RNAPI-associated nascent transcripts (Figures 4D, 4E, S4C, and S4D). The fraction of RNAPI-associated reads that carried non-templated oligo(A) sequences (AAA or longer) was highest at sites of slowest elongation (Figure 4D). The effects were strongest over the 5′ ETS (Figures 4D and 4E) but were visible across the entire rDNA (Figures S4C and S4D). We conclude that the nascent transcript can be adenylated while bound to RNAPI and that this is most common at sites where elongation is slowest and prone to backtracking.

In the elongating complex, the 3′ end is sequestered within the polymerase. This suggested adenylation might be specific for stalled and backtracked RNAPI, from which the 3′ end may be extruded, as demonstrated for RNAPII.[51] To test whether the TRAMP complex can adenylate the nascent transcript associated with backtracked polymerase, we reconstituted this activity *in vitro* (Figures 4F, 4G, and S4E). The TRAMP4 (Trf4 plus Air1/2) complex was purified from yeast using Trf4-TAP. Elongating RNAPI was purified using Rpa135-HTP as above and incubated with the DNA template shown in Figure S3. RNAPI was stalled at the G9 tract and allowed to backtrack by NTP washout. Incubation with TRAMP4 plus ATP resulted in progressive elongation of the nascent transcript (Figures 4G and S4E). When the analysis was repeated using RNAPI-Rpa12ΔC, accumulation of backtracked RNA was more pronounced, as expected, but less processive adenylation was observed (Figure S4E).

**Figure 3. Efficient termination requires exonuclease activity by Rat1 and endonuclease activity of RNAPI**

(A) Schematic of the *in vitro* termination assay. A description is given in the text. For a detailed version, see Figure S3.

(B) *In vitro* termination assay. The western blot shows Rpa135-HTP (RNAPI) distribution between the DNA-bound (B) fraction and the supernatant (S) that represents terminated and released RNAPI.

(C) Quantitation of data in (B). Some release of RNAPI from the template DNA was already observed at T0, so this initial level was set to 0. The subsequent increase in RNAPI release over time is indicated by the graphs. Complete release is 100%. See methods for the details. Data are represented as mean ± SD. *$p < 0.05$, two-tailed t test, $n = 5$).

(D) Two potential mechanisms participating in torpedo termination of RNAPI: (1) Rat1-mediated mechanism and (2) backtracking-mediated mechanism.

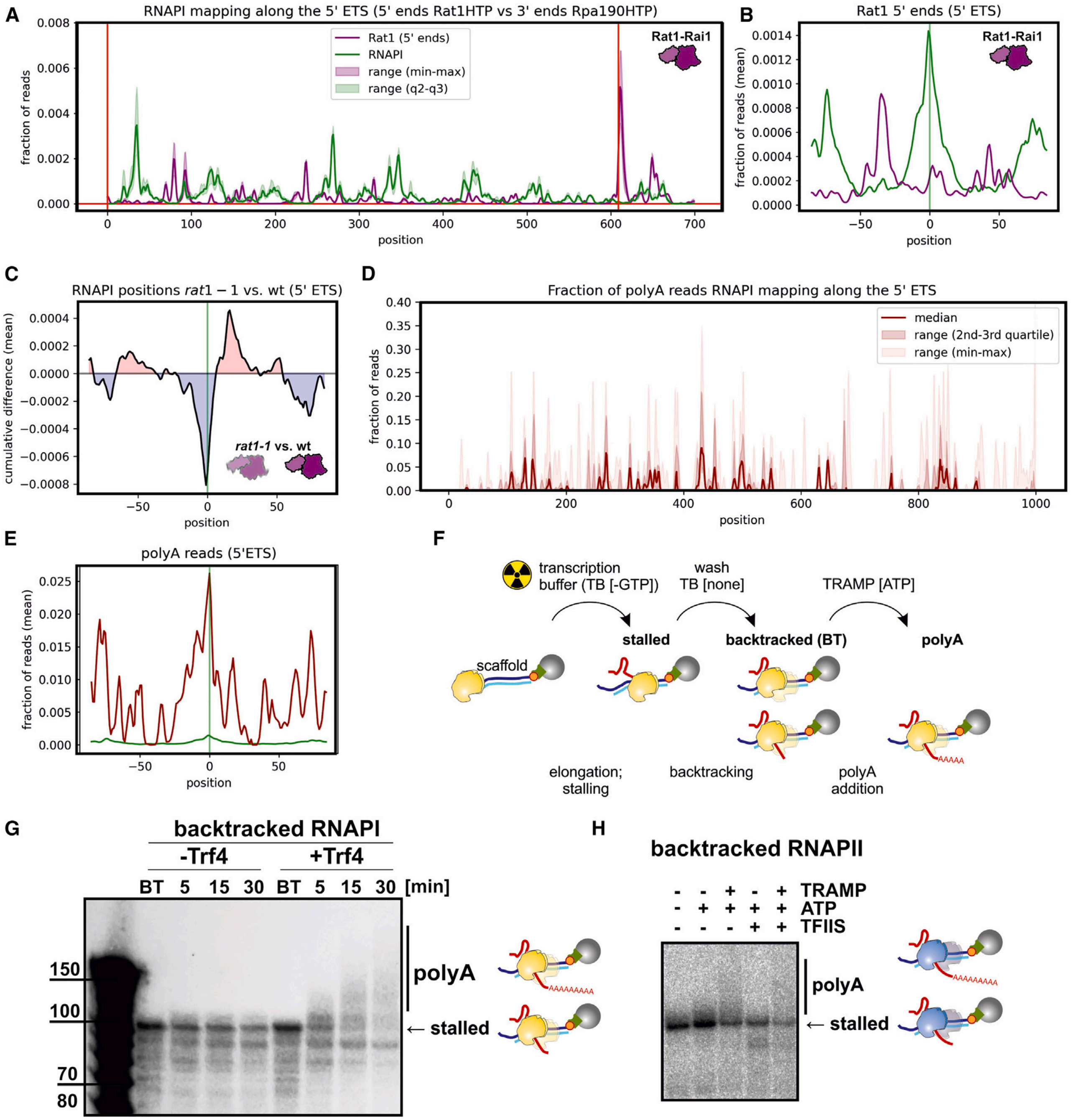


**Figure 4. Rat1 and TRAMP are found at sites of slowed RNAPI elongation**

(A) Rat1 associated with sites of slowed/paused RNAPI. The 5′ ends of Rat1-HTP mapped reads are presented. Data are represented as mean ($n$ = 2).

(B) RNAPI (Rpa190-HTP) and Rat1 peak metaplots across the rDNA (*RDN37*). The +643 peak was excluded from this analysis (see also Figure S4B).

(C) Cumulative difference map for RNAPI (Rpa190-HTP) in *rat1-1* versus WT. The *rat1-1* mutation leads to rearrangements of RNAPI-associated peaks within the 5′ ETS region. The RNAPI peaks are less prominent and shifted in *rat1-1* mutant when compared to WT cells.

(D) Fraction of oligo(A)+ reads recovered in RNAPI CRAC data (non-coded $A_n \geq 3$), mapped across the 5′-ETS. Data are represented as median ($n$ = 6).

(E) RNAPI CRAC peak metaplot for 5′ ETS, comparing the 3′ ends of the reads (green) with poly(A) reads (maroon). As expected, the Rpa190 distribution matches the metapeak plot giving a low signal.

(F) Schematic of the *in vitro* assay for transcription, backtracking, and adenylation.

*(legend continued on next page)*

The same assay was applied to RNAPII purified with TAP-tagged Rpb1 (Rpo21). We observed adenylation of the backtracked nascent RNAPII transcript in the presence of purified TRAMP4 (Figure 4H). For RNAPII, the homolog of Rpa12 is TFIIS (Dst1 in yeast), which is not a stable component of the polymerase holoenzyme. Addition of purified TFIIS reduced backtracked RNA accumulation and adenylation (Figure 4H).

To assess whether poly(A) tails were also added by the TRAMP complexes *in vivo*, we initially assessed Mtr4 binding. We mapped the 3′ ends, selecting only oligo-adenylated reads (sequence data from GSE77863), which are expected to reflect sites of TRAMP activity.[52,53] This analysis was performed in a strain expressing Rrp44-exo,[54] lacking the exonuclease activity of Rrp44, to stabilize transient intermediates of degradation. RNAPI peaks overlapped with peaks of $p(A)^+$ reads recovered with Mtr4 (Figure 5A), and this was supported by metaprofile analysis (Figure 5B). Comparison of RNAPI with all Mtr4 reads is shown in Figure S5A. Mtr4 also has functions that do not require other, partially redundant components of the TRAMP complex (Trf4 or Trf5 plus Air1 or Air2). We therefore tested the effects of the *trf4-1 trf5Δ* and *air1Δ air2Δ* double mutants, which are expected to abolish TRAMP activity, on Rpa190 distribution and nascent transcript adenylation (Figures 5C–5F). In both double-mutant strains, the size of RNAPI peaks was strongly altered (Figures 5C and 5D), and nascent transcript adenylation was blocked almost completely (Figures 5E and 5F). At the sites called as peaks in the WT, the signals were reduced in the mutants. Note, however, that in normalized sequencing data the same total number of reads is distributed for each sample, and we interpret this result as indicating that pausing occurs at more sites in the mutant strains. From these data, we conclude that backtracked, nascent transcripts associated with stalled RNAPI are targets for the TRAMP complex.

Since Rpa12 endonuclease activity was implicated in termination in the 3′-ETS, we tested the effects of *rpa12ΔC* on RNAPI occupancy using Rpa135-HTP (Figure S5B). Only differences in peaks were seen for *rpa12ΔC* relative to the WT. We also assessed the effects of Rrp44-exo, which showed a more uniform distribution of RNAPI (Rpa190-HTP) along the 5′ ETS relative to the WT, as seen in metaprofile analysis (Figure S5C). The double mutant *rpa12ΔC rrp44-exo* showed apparent accumulation of RNAPI within the 5′ ETS region (Figures 5G and S5D). Notably, the accumulation occurred at different positions for the WT (+437 and +647; Figure S5B) and Rrp44-exo (+36, +125, and +195; Figures 5G and S5D) strains, indicating differential sensitivity of RNAPI to stalling in these backgrounds.

We speculate that Rrp44 activity plays a role in establishing RNAPI distribution along the 5′ ETS (refer to the discussion on the "reverse torpedo" model for details). Increased densities at specific pause sites in *rpa12ΔC* support the model that Rpa12 contributes to RNAPI release from pausing.

These data indicate that a significant level of transcriptional pausing by RNAPI occurs within the 5′ ETS region. Upstream polymerases will be blocked by the stall. In addition, since transcribing polymerases are expected to be strongly entrained, downstream polymerases may also be torsionally stalled, potentially imposing considerable strain on the DNA. Human Top2A binds RNA, with specificity for 3′ ends carrying 3′ OH groups, as would be the case for backtracked nascent transcripts.[55] Yeast Top2 was identified in a screen for RNA-interacting proteins using UV crosslinking followed by density centrifugation (identification of RNA-associated peptides).[56,57] We therefore speculated that Top2 might be recruited to the 3′ end of the nascent pre-rRNA at sites of RNAPI backtracking to relieve torsional stress. A Top2-HTP strain showed no clear growth defects, indicating that the fusion protein is functional, and gave good signals in CRAC, confirming *in vivo* RNA association. Mapping Top2-HTP crosslinking sites on the rDNA revealed peaks of occupancy in the 5′ ETS that are adjacent to sites of RNAPI pausing/stalling (Figure 5H). This was supported by metaprofile analyses (Figures S5E and S5F). Notably, we recovered oligo(A) tailed, Top2-associated reads, and these mapped closely with RNAPI-associated $oligo(A)^+$ reads (Figure 5I). This strongly supports the association of Top2 with backtracked nascent transcripts and reaffirms the conclusion that these adenylated pre-rRNA fragments are chromatin associated.

We propose that Top2 is recruited to stalled, backtracked RNAPI due to accumulation of supercoiled DNA and acts to "break" the torsional entrainment, allowing downstream polymerases to continue transcription. The functional significance of RNA binding is unclear, but recovery of many oligo(A) reads indicates that Top2 is recruited to stalled and backtracked RNAPI.

## *In silico* simulation of premature termination and transcriptional output

The CRAC data strongly suggested that pre-rRNA transcription is subject to a degree of premature termination, but the termination frequency cannot be accurately determined from these data alone. To quantitatively estimate the range of premature termination, we modified a previously developed computational model for RNAPI transcription.[7] This model incorporates various factors that affect transcription elongation, including stochastic elongation rate, DNA torsion and RNAP entrainment, stability of the transcription bubble within RNAPI, and the impact of the extruded nascent RNA on backtracking. These parameters are described in detail in our previous publication.[7]

To estimate the range of RNAPI transcriptional processivity consistent with rRNA production *in vivo*, we incorporated literature values. These include 75 transcriptionally active rDNA repeats (50% of the 150 total rDNA repeats),[58] an average of 50 RNAPIs per active rDNA repeat,[7,59] and the production of 200,000 newly synthesized ribosomes per cell per generation (100 min),[60] although this represents the higher end of the growth rate range.[61]

We aimed to incorporate different levels of RNAPI processivity into the model and simulated for 6,000 s (100 min), reflecting

(G) Purified Trf4-Air1/2 can add poly(A) tails to nascent RNA associated with backtracked RNAPI. Transcripts were internally radiolabeled and visualized with an image analyzer (Fuji-Film).
(H) Trf4 oligo-adenylates the 3′ end of backtracked, nascent RNA *in vitro* extruded from RNAPII. The assay was performed as outlined in (F) but using purified RNAPII and TFIIS in addition to Trf4-Air1/2.

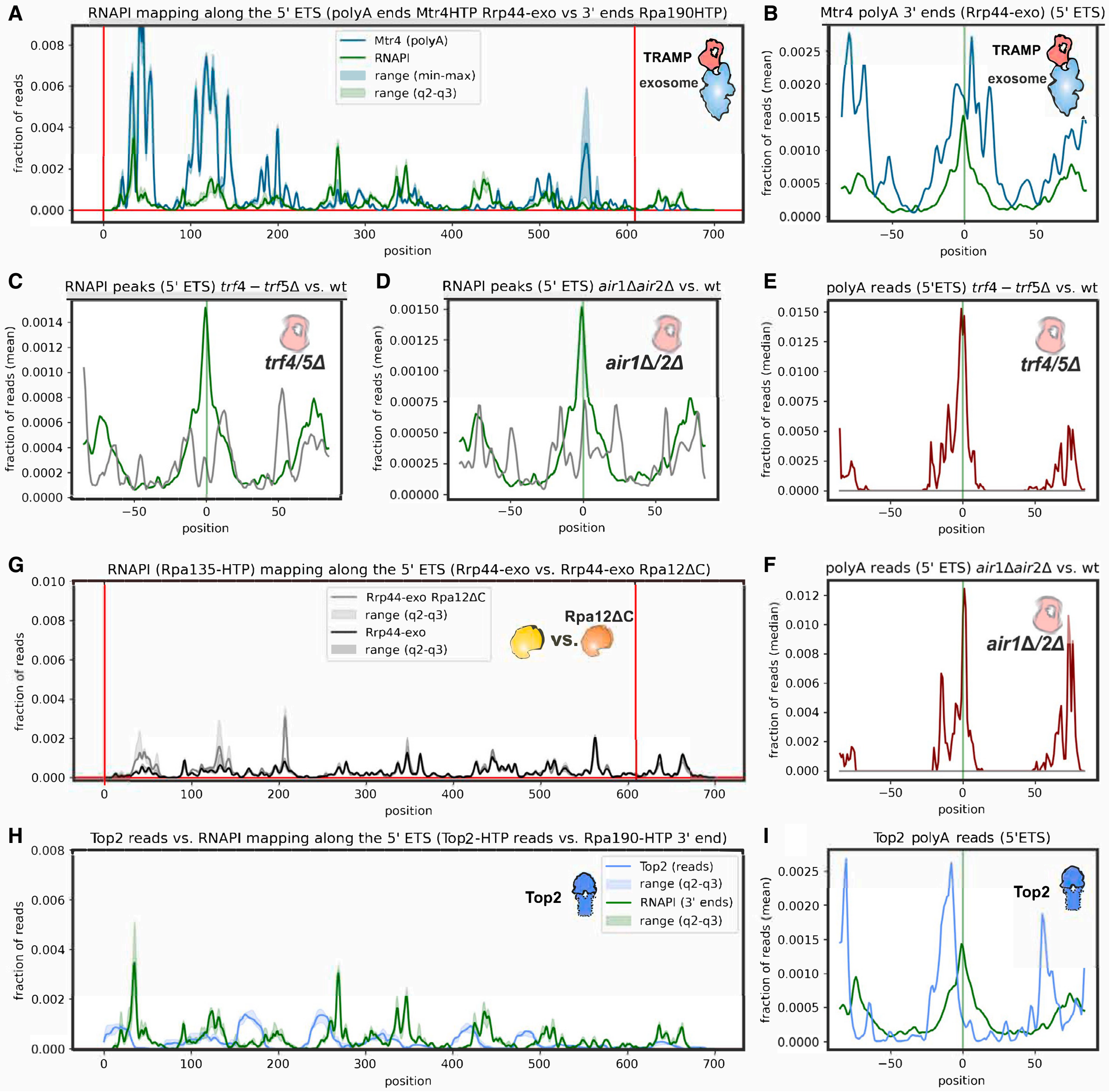


**Figure 5. Nascent transcripts can be oligo-adenylated by the TRAMP complex**

(A) Adenylated ($A_n \geq 3$) Mtr4 CRAC reads (blue) superimposed on RNAPI (Rpa190-HTP) 3′ end reads (green). Analyses were performed in rrp44-exo strains, which lack a major exonuclease activity of the exosome complex. Data are represented as median (*n* = 6) for Rpa190-HTP and mean for Mtr4-HTP rrp44-exo (*n* = 2).

(B) Metaplot of adenylated ($A_n \geq 3$) Mtr4 CRAC reads (blue) superimposed on RNAPI (Rpa190-HTP) 3′ end reads (green).

(C and D) RNAPI (Rpa190-HTP) peaks within the 5′ ETS region (green lines) are reduced and more heterogeneous in *trf4-1 trf5Δ* and *air1Δ air2Δ* double-mutant backgrounds (gray lines).

(E and F) RNAPI-associated poly(A) reads in the WT (dark red line) are absent in *trf4-1 trf5Δ* (and *air1Δ air2Δ* (gray lines) strains. Note that the lines for the mutants lie along the x axis. For clarity, we display the median of reads in this analysis.

(G) RNAPI (Rpa135-HTP) distribution across the 5′ ETS, comparing WT (black) with rpa12ΔC (gray) strains. Analyses were performed in rrp44-exo strains, which lack a major exonuclease activity of the exosome complex. Data are represented as median (*n* = 3).

(H) Distribution of Top2-HTP CRAC reads across the 5′ ETS superimposed on RNAPI (Rpa190-HTP) 3′ ends (green). Data are represented as median (Top2-HTP, *n* = 5; Rpa190-HTP, *n* = 6).

(I) Metaplot of 3′ ends of adenylated ($A_n \geq 3$) Top2 reads (blue) across the 5′ ETS superimposed on RNAPI (Rpa190-HTP) 3′ ends (green).

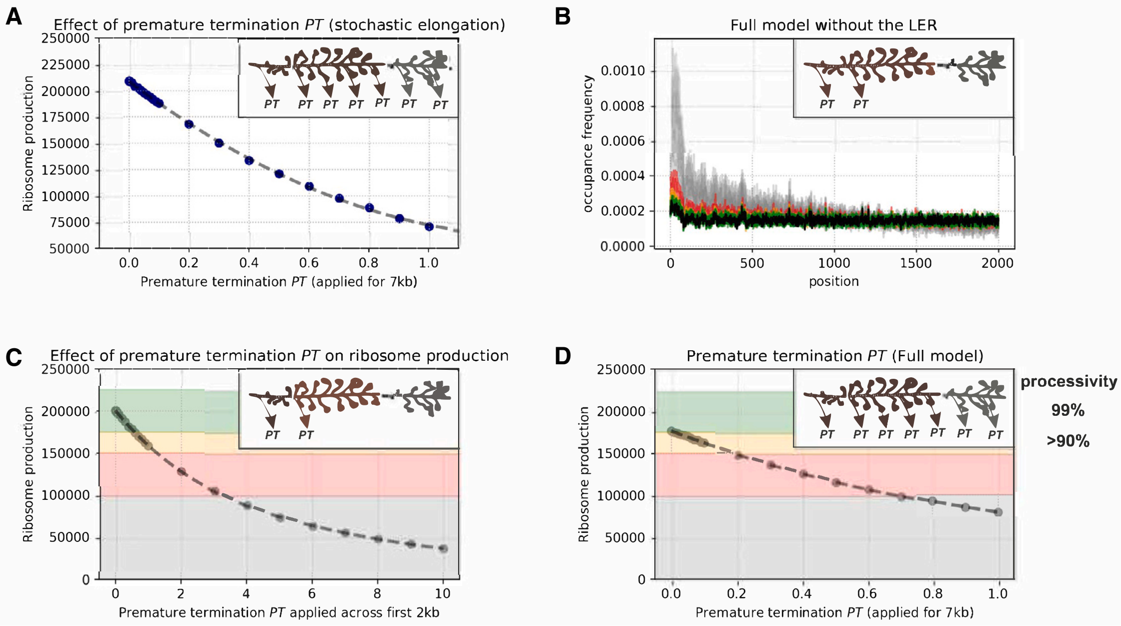


**Figure 6. The computational model of RNAPI transcription predicts range of premature termination *in vivo***

(A) Effect of premature termination (PT) on ribosome production in a model where RNAPI velocity is driven by stochastic elongation only. PT was applied across the entire transcription unit.

(B) RNAPI occupancy profiles after application of PT. Profiles are superimposed and color coded to correspond with the background in (C): black, no premature termination PT; green and orange, PT where ribosome synthesis is effective; red, ribosome synthesis is decreased (<150,000 per generation); gray, ribosome synthesis is insufficient (<100,000 per generation).

(C) Effects of PT on ribosome production in a model including stochastic elongation, RNA elements, and DNA torsion forces. PT was applied across the initial 2 kb of the transcription unit. Background colors match the color codes in (B). Since PT is applied only across a region of the transcription unit, values are significantly higher than for (A) and (C).

(D) Effect of premature termination PT on ribosome production. 10% of RNAPI can terminate prematurely without significant impairment of overall ribosome biosynthesis.

one yeast cell division. From the output of 75 repeats, we calculated the total number of fully synthesized pre-rRNA molecules ($RNAP_{productive}$), the number of prematurely terminated pre-rRNAs ($RNAP_{non-productive}$), processivity, and the average number of RNAPIs per single rDNA repeat (RNAPI number). The initial results highlighted that the premature termination rate cannot be equated directly with processivity, as defined by the ability of RNAPI to reach the end of the transcription unit:

$$processivity = \frac{RNAP_{productive}}{RNAP_{productive} + RNAP_{non-productive}}.$$

RNAP elongation is a stochastic process that follows a Brownian ratchet mechanism, resulting in a non-uniform distribution of occupancy time due to multiple factors that affect the velocity. We anticipate that premature termination is related to the occupancy time at a particular position rather than being equally distributed throughout the transcription unit. This is supported by the observation that low RNAPI velocity (indicating high occupancy) was strongly correlated with RNA transcript binding by the surveillance machinery (Figure 4).

To account for this, we incorporated the probability of premature termination ($P_{PT}$) in our model, which is scaled according to the estimated total transcription time. This approach considers the probability of premature termination at each position along the transcription unit, providing a more accurate representation of the stochastic nature of the transcription process:

$$P_{PTadj} = \frac{P_{PT}}{time}, \text{where } time \approx \frac{length}{\overline{V}}, \text{and}$$

$\overline{V}$ is average velocity. In our implementation, the value of $P_{PT}$ approximates to *processivity*, although it does not fully satisfy its definition. By incorporating $P_{PT}$ as a parameter in our model, we consider the likelihood of premature termination at each position along the transcription unit, which provides insight into the stochastic nature of the elongation process. However, $P_{PT}$ does not directly measure the RNAP ability to reach the end of the transcription unit, which is the definition of *processivity*. Therefore, although $P_{PT}$ and processivity share some similarities, they are not equivalent measures of RNAP transcription.

We conducted a series of simulations to test the implementation of premature termination as $P_{PT}$, using a stochastic RNAP elongation mechanism and an initiation probability of 0.8. The previously published model optimized the transcription initiation probability at 0.8, giving the best fit to the biological output.[7] We calculated the number of rRNA molecules produced and RNAPI molecules per rDNA and investigated how $P_{PT}$ value influenced RNAPI transcriptional output (Figure 6A). We have proposed previously that the observed 5′ bias in rDNA transcription

originates from the progressive entrainment of multiple RNAPI molecules across the transcription unit, with low-entrainment regions (LERs) close to the TSS[7] (Figures S6A–S6C). To validate this model, we included $P_{PT}$ in the simulations across the first 2,000 nt where 5′ bias was observed (Figures 6B, 6C, and S6D–S6G).

Including $P_{PT}$ in the absence of the LERs reproduced the observed 5′ bias (Figure 6B) but led to significantly reduced ribosome production (Figure 6C); inconsistent with the demand for 200,000 ribosomes per generation. $P_{PT}$ values below 0.2 were required to produce numbers of ribosomes consistent with the reported range (Figure 6D). Values above 0.7 are required to generate the observed excess of polymerases in the 5′ region of the rDNA. RNAPI accumulation at the 5′ end of the rDNA is therefore unlikely to be solely due to premature termination.

We also observed a significant level of polymerase pausing, which can break the convoy of entrained polymerases and lead to runoff. However, polymerases located 5′ will be blocked, and any RNAPI complex that remains stalled will eventually be ubiquitinated and degraded off the DNA.[62,63] Therefore, the overall frequency of premature termination by RNAPI is unlikely to exceed 20%, but most likely values are around 10%. While this proportion is low, cumulatively, 10,000 transcription events may be terminated prematurely per generation (~100 events/min). At steady state, the cellular abundance of abortive transcripts is likely to be low due to efficient clearance by the surveillance machinery.

## DISCUSSION

Eukaryotic transcription elongation rates show considerable heterogeneity with functional consequences for the many RNA processing and packaging factors acting on the nascent transcript. RNAPI transcribes a single pre-rRNA transcript from the nucleosome-free rDNA, making it well suited to mechanistic analyses. We previously observed 5′ enrichment for RNAPI density with strikingly uneven, local polymerase distribution, most notably over the 5′ ETS region of the pre-rRNA (Figure 1).

In the 3′-ETS the RNAPI-associated RNAs showed a strong peak of 5′ ends at the 3′ cleavage site reported for Rnt1 (B0-2). No clear peak was seen for the 5′ cleavage site, consistent with the expectation that Rnt1, like other RNase III-related enzymes, cleaves across the stem. RNA fragments with 5′ ends at site B0-2, showed a strong bias in length distribution, with 3′ ends greatly favored at position +25 nt. This indicates very rapid co-transcriptional Rnt1 cleavage across the terminal stem, when the polymerase has progressed only 25 nt, with 10–12 nt of extruded nascent transcript. This speed was initially surprising, but we note that pre-mRNA splicing can be 50% complete when RNAPII has advanced only 45 nt.[64] At least in part, this rapidity reflects coupling of slowed/paused RNAPII elongation with splicing.[65,66] For RNAPI, we postulate that release of the nascent pre-ribosome by Rnt1 cleavage relieves the polymerase of torsional entrainment. This confers increased freedom to rotate with the DNA rather than continuing to elongate. Rnt1 depletion greatly reduced the RNAPI signal at B0, as expected, but also reduced the downstream peak of 3′ ends, supporting linkage between co-transcriptional cleavage and elongation pausing. This deceleration is very likely important in facilitating Rat1-mediated termination. For RNAPII, deceleration following co-transcriptional cleavage makes it a "sitting duck" for Rat1 (Xrn2) termination.[67]

Our data also revealed that endonuclease cleavage by Rpa12 facilitates effective termination, presumably acting on backtracked transcripts. During torpedo termination, the nascent transcript must be extracted from the polymerase, and cleavage of backtracked transcripts may facilitate this process. The DNA binding protein Nsi1 has been reported to contribute to termination by binding downstream of the major terminator site T1.[24–26] We observed increased readthrough in *nsi1Δ* strains, but the extent was modest, and the major peak adjacent to the Rnt1 cleavage site was not clearly altered. Termination may normally be promoted by the interplay between entrainment release following Rnt1 cleavage, roadblocking by Nsi1, and the Rat1 torpedo activity, with Rpa12 cleavage aiding release of backtracked polymerases.

A significant level of TSS proximal termination was also identified. We previously reported excess RNAPI and very uneven density within the 5′ ETS.[7] We concluded that, as for the 3′ ETS, short nascent transcripts in this region reduce entrainment, facilitating pausing and backtracking, particularly at sites of weak folding in the nascent transcript. At these locations, RNAPI is associated with oligo(A)-tailed RNAs, indicating 3′ adenylation of backtracked nascent transcripts. Consistent with this, the Mtr4 component of TRAMP was localized to transcription pause sites. Moreover, we could reproduce TRAMP-mediated adenylation of nascent transcripts on backtracked RNAPI *in vitro*. Loss of TRAMP activity abolished nascent transcript adenylation and was associated with strongly altered RNAPI peak distribution. We initially anticipated that peaks would be enhanced by the mutations, but this was not what we found. Rather, we observed density peaks at additional locations, indicating more heterogeneous transcriptional pausing. We postulate that the very prominent peaks in the WT represent the most stable, or least readily resolved, stall sites. In the mutants, polymerases are stabilized at additional pause sites. As total occupancy in the normalized data remains constant, this is seen as a reduction in the largest peaks relative to the WT. These observations support the model that backtracked RNAPI is targeted and oligo-adenylated by TRAMP, a key cofactor for the exosome nuclease complex. We predict that the exosome degrades the 3′-extruded pre-rRNAs, potentially promoting termination by a reverse torpedo mechanism (Figure 7). For RNAPII, pausing and backtracking is common (for examples, see previous studies[4–6,43,44,51]), and we were able show adenylation of the backtracked, RNAPII-associated, nascent transcript, suggesting that the mechanism may be common. The 5′ exonuclease Rat1 was localized immediately upstream of the TSS-proximal RNAPI pause sites. A point mutation in Rat1 was associated with altered RNAPI peak distribution with increased heterogeneity. We interpret this as indicating that "conventional" torpedo termination also occurs at these locations.

We cannot accurately determine the relative contributions of forward and reverse torpedo mechanisms to premature termination, but the presumed intermediates in each pathway were detected in unperturbed cells, indicating that both normally

CellPress
OPEN ACCESS

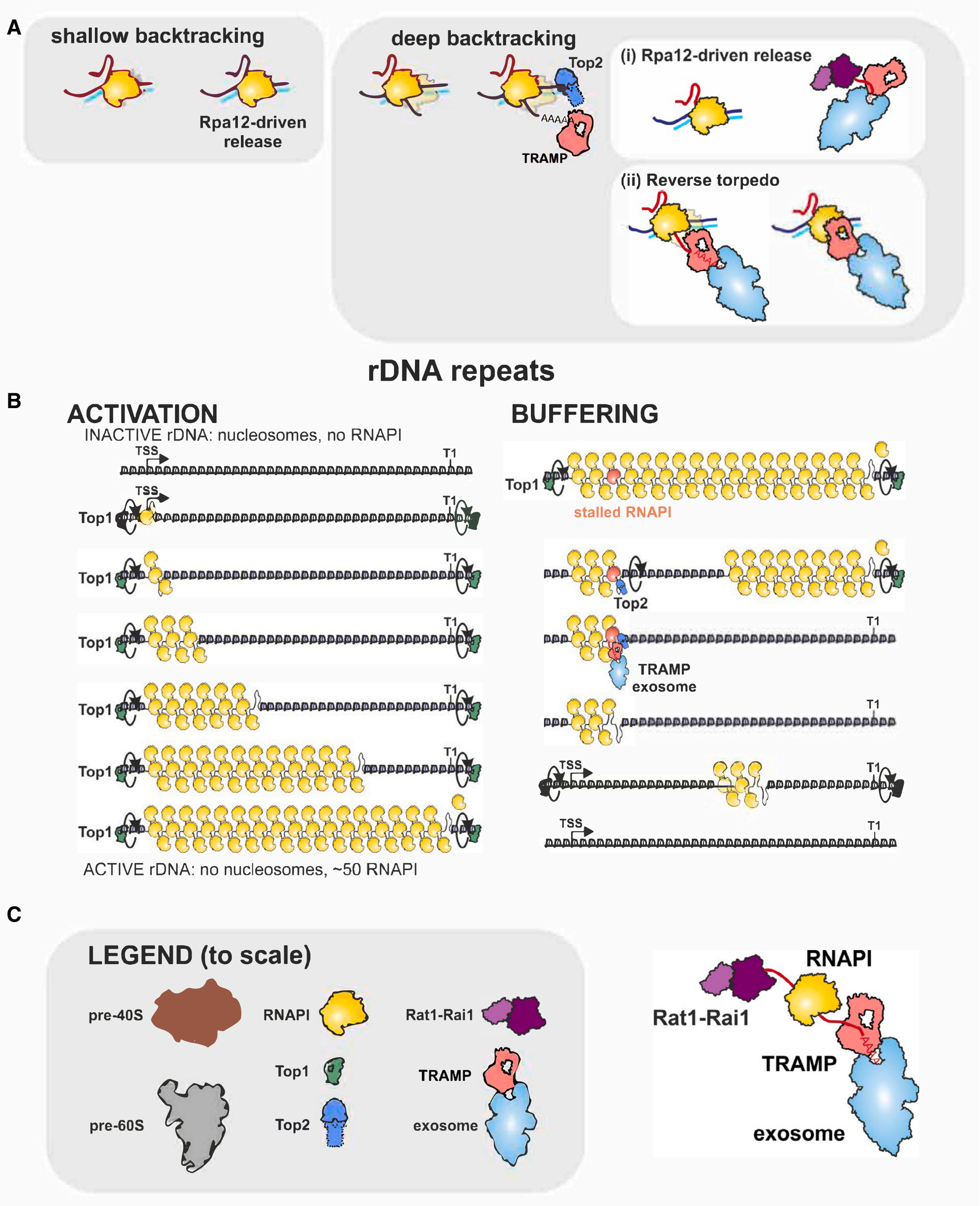


**Figure 7. Models for RNAPI transcription**

(A) Shallow backtracking can be released by Rpa12-mediated cleavage of the nascent transcript. We propose that release from deep backtracking is initiated with oligo-adenylation of the nascent transcript by the TRAMP complex and promoted by (1) Rpa12-driven release and/or (2) a reverse torpedo mechanism.

(B) Left: inactive rDNA repeats are packaged in nucleosomes but can be opened by the cooperative activities of multiple polymerases, each linked by torsional entrainment. Right: a single stalled or deeply backtracked RNAPI will break the convoy of polymerases. Continued transcription by downstream polymerases can be facilitated by Top2 recruitment. The stalled polymerase may be released by combined torpedo (Rat1) and reverse torpedo (TRAMP, exosome) activities. Relative numbers and sizes of nucleosomes and RNAPI complexes are approximately correct and consistent with the length of the rDNA. Nascent pre-ribosomes would be very much larger in size but are omitted for clarity.

(C) Model components are summarized to scale, plus the forward and reverse torpedoes.

participate. Biophysical modeling is consistent with around 10% of pre-rRNAs transcription terminating in the 5′ ETS. Given the very high rates of ribosome synthesis in yeast, this represents a substantial number of events (around 100 per minute per cell).

## Cycle of rDNA transcription

In both yeast and human cells, only around half of the rDNA repeats are actively transcribed at any time.[57] The basis for this apparent excess has been unclear, especially in yeast with its compact genome, in which the rDNA is around 10% of the total. We postulate that this may be related to the finding of substantial polymerase stalling described here. A single stalled polymerase will break the convoy of coordinately transcribing polymerases on the rDNA, potentially blocking elongation by all polymerases on the transcription unit (Figure 7).

Shallow RNAPI backtracking (up to 20 nt) can be resolved by the intrinsic endonucleolytic activity of Rpa12 within RNAPI. However, recovery from deep RNAPI backtracking is more limited,[68] and we propose that this is promoted by (1) Rpa12-driven release followed by TRAMP-mediated pull of the 3′ nascent RNA and/or (2) by the reverse torpedo mechanism (Figure 7A). If a single polymerase stalls or backtracks, then the downstream polymerases, still torsionally entrained, will be moving away at ∼40 nt/s. So, a second later, around 4 negative supercoils will already have accumulated in front of the stalled polymerase, which can be accommodated by either “writhe” (twisting) or strand separation. We note that the combination of negative supercoils and polymerase backtracking will be conducive to anterior R-loop formation. Consistent with this model, we previously noted a high level of R-loop formation on the rDNA 5′ ETS.[69] With extended pausing, the resulting high torsion will potentially lead to DNA damage. The RNA interactions of Top2 indicated association with nascent transcripts, particularly oligoadenylated RNAs indicative of sites of polymerase backtracking. Recruitment of Top2 to the backtracked polymerases would allow runoff for downstream polymerases, reducing the torsional burden. The stall could then be cleared through termination or, failing that, via ubiquitination and proteasome-mediated degradation of the “broken” polymerase.[62,63]

We postulate that the loss of transcription activity on a single rDNA unit is followed by its closure and packaging into a chromatin array of nucleosomes, accompanied by opening of previously closed repeats to maintain active rDNA repeat numbers. Activation of a repeat requires transcription through the nucleosome array. Initially by a single RNAPI, as more polymerases are loaded, they will increasingly act cooperatively as a convoy linked by DNA torsion. Transcription by RNAPII through nucleosomes is facilitated by numerous cofactors,[70] most of which are not shared by RNAPI (but see Schneider et al.[71]). These are required, in part, to re-establish nucleosomes following polymerase passage. However, actively transcribed rDNA regions are believed to be nucleosome free, as indicated in Figure 7. The pioneer RNAPI, aided by all subsequent polymerases, only needs to displace nucleosomes from the DNA. Nucleosomal DNA is negatively supercoiled, so the positive supercoils expected in front of the leading polymerase may promote nucleosome displacement, facilitating clearance of the transcription unit.

Finally, we note that the presence of large numbers of inactive, but intact and potentially functional, repeats has been maintained over long evolutionary timescales. The data presented here suggest that the active repeats are prone to blockage and inactivation, potentially by a single stalled RNAPI plus a pre-ribosome complex. We postulate that, when this arises, previously inactive repeats can be rapidly derepressed to maintain the correct ribosome synthesis rate.

## Limitations of the study

This study reveals sites and potential mechanisms of transcription termination by RNAPI. While we provide estimates of the premature termination rate for RNAPI transcription, we advise caution when drawing quantitative conclusions about the elements of the RNAPI termination systems. Specifically, this study does not calculate the relative contributions of different elements in the unperturbed state; these include Rat1-dependent torpedo termination, backtracking-facilitated torpedo termination, Nsi1 roadblock, and the reverse torpedo mechanism.

## RESOURCE AVAILABILITY

### Lead contact

Requests for further information, resources, and reagents should be directed to and will be fulfilled by the lead contact, David Tollervey (d.tollervey@ed.ac.uk).

### Materials availability

All yeast strains generated during this project are freely available from the lead contact.

### Data and code availability

- All CRAC sequencing data have been deposited at GEO: GSE246546 and are publicly available as of the date of publication.
- All original code has been deposited at Zenodo at https://doi.org/10.5281/zenodo.14592083 (data reanalysis pipeline and notebooks) and https://doi.org/10.5281/zenodo.14587255 (mathematical model) and is publicly available as of the date of publication. The analysis was conducted using trxtools v.0.2.1 (https://github.com/TurowskiLab/trxtools).
- Any additional information required to reanalyze the data reported in this paper is available from the lead contact upon request.

## ACKNOWLEDGMENTS

We thank Olivier Gadal and Christophe Dez (Centre de Biologie Intégrative, Toulouse) for critical reading of the manuscript and Jan Mikołajczyk (IBB PAS) for stimulating discussions. T.W.T. and K.G.-N. were supported by the Polish National Agency for Academic Exchange (PPN/PPO/2020/2/00004/U/00001) and National Science Center (2020/39/D/NZ2/02115). M.-L.W. was supported by an EMBO Long Term Fellowship (ALTF 348-2015). D.T. and E.P. were supported by Wellcome Principal Research Fellowships (109916 and 222516).

## AUTHOR CONTRIBUTIONS

T.W.T. and D.T. conceived the project and wrote the manuscript. E.P., M.-L.W., and K.G.-N. performed experiments. T.W.T. and D.T. developed the mathematical model. All authors analyzed the data. All authors edited and reviewed the manuscript.

## DECLARATION OF INTERESTS

The authors declare no competing interests.

CellPress
OPEN ACCESS

## STAR★METHODS

Detailed methods are provided in the online version of this paper and include the following:

- KEY RESOURCES TABLE
- EXPERIMENTAL MODEL AND STUDY PARTICIPANT DETAILS
  - Strains
- METHOD DETAILS
  - *In-vivo* RNA crosslinking
  - CRAC
  - Tap purification of Rat1, Rai1 and Trf4
  - Purification of RNA polymerase I and *in vitro* assay
  - Polyadenylation assays
  - Termination assays
  - Modification of mathematical model for RNAPI transcription
- QUANTIFICATION AND STATISTICAL ANALYSIS
  - Analysis reproducibility
  - Pre-processing and data alignment
  - Selection of the 5′-ends, 3′-ends and polyA
  - RNA polymerase I profile
  - Peak calling and metaplots
  - Statistical analyses and numerical methods
- ADDITIONAL RESOURCES

## SUPPLEMENTAL INFORMATION

Supplemental information can be found online at https://doi.org/10.1016/j.celrep.2025.115325.

Received: January 3, 2024
Revised: November 1, 2024
Accepted: January 28, 2025
Published: February 24, 2025

## REFERENCES

1. Ma, J., Bai, L., and Wang, M.D. (2013). Transcription Under Torsion. Science *340*, 1580–1583. https://doi.org/10.1126/science.1235441.
2. Dangkulwanich, M., Ishibashi, T., Liu, S., Kireeva, M.L., Lubkowska, L., Kashlev, M., and Bustamante, C.J. (2013). Complete dissection of transcription elongation reveals slow translocation of RNA polymerase II in a linear ratchet mechanism. Elife *2*, e00971. https://doi.org/10.7554/eLife.00971.
3. Guajardo, R., and Sousa, R. (1997). A model for the mechanism of polymerase translocation. J. Mol. Biol. *265*, 8–19. https://doi.org/10.1006/jmbi.1996.0707.
4. Noe Gonzalez, M., Blears, D., and Svejstrup, J.Q. (2021). Causes and consequences of RNA polymerase II stalling during transcript elongation. Nat. Rev. Mol. Cell Biol. *22*, 3–21. https://doi.org/10.1038/s41580-020-00308-8.
5. Sheridan, R.M., Fong, N., D'Alessandro, A., and Bentley, D.L. (2019). Widespread Backtracking by RNA Pol II Is a Major Effector of Gene Activation, 5′ Pause Release, Termination, and Transcription Elongation Rate. Mol. Cell *73*, 107–118.e4. https://doi.org/10.1016/j.molcel.2018.10.031.
6. Rodríguez-Molina, J.B., West, S., and Passmore, L.A. (2023). Knowing when to stop: Transcription termination on protein-coding genes by eukaryotic RNAPII. Mol. Cell *83*, 404–415. https://doi.org/10.1016/j.molcel.2022.12.021.
7. Turowski, T.W., Petfalski, E., Goddard, B.D., French, S.L., Helwak, A., and Tollervey, D. (2020). Nascent transcript folding plays a major role in determining RNA polymerase elongation rates. Mol. Cell *79*, 488–503.e11. https://doi.org/10.1016/j.molcel.2020.06.002.
8. Brill, S.J., DiNardo, S., Voelkel-Meiman, K., and Sternglanz, R. (1987). Need for DNA topoisomerase activity as a swivel for DNA replication for transcription of ribosomal RNA. Nature *326*, 414–416. https://doi.org/10.1038/326414a0.
9. Krawczyk, C., Dion, V., Schär, P., and Fritsch, O. (2014). Reversible Top1 cleavage complexes are stabilized strand-specifically at the ribosomal replication fork barrier and contribute to ribosomal DNA stability. Nucleic Acids Res. *42*, 4985–4995. https://doi.org/10.1093/nar/gku148.
10. Christman, M.F., Dietrich, F.S., Levin, N.A., Sadoff, B.U., and Fink, G.R. (1993). The rRNA-encoding DNA array has an altered structure in topoisomerase I mutants of Saccharomyces cerevisiae. Proc. Natl. Acad. Sci. USA *90*, 7637–7641. https://doi.org/10.1073/pnas.90.16.7637.
11. El Hage, A., French, S.L., Beyer, A.L., and Tollervey, D. (2010). Loss of Topoisomerase I leads to R-loop-mediated transcriptional blocks during ribosomal RNA synthesis. Genes Dev. *24*, 1546–1558. https://doi.org/10.1101/gad.573310.
12. Schärfen, L., and Neugebauer, K.M. (2021). Transcription Regulation Through Nascent RNA Folding. J. Mol. Biol. *433*, 166975. https://doi.org/10.1016/j.jmb.2021.166975.
13. Kos, M., and Tollervey, D. (2010). Yeast pre-rRNA processing and modification occur cotranscriptionally. Mol. Cell *37*, 809–820. https://doi.org/10.1016/j.molcel.2010.02.024.
14. Vogelauer, M., and Camilloni, G. (1999). Site-specific in vivo cleavages by DNA topoisomerase I in the regulatory regions of the 35 S rRNA in Saccharomyces cerevisiae are transcription independent. J. Mol. Biol. *293*, 19–28. https://doi.org/10.1006/jmbi.1999.3154.
15. Di Felice, F., Cioci, F., and Camilloni, G. (2005). FOB1 affects DNA topoisomerase I in vivo cleavages in the enhancer region of the Saccharomyces cerevisiae ribosomal DNA locus. Nucleic Acids Res. *33*, 6327–6337. https://doi.org/10.1093/nar/gki950.
16. Di Felice, F., Egidi, A., D'Alfonso, A., and Camilloni, G. (2019). Fob1p recruits DNA topoisomerase I to ribosomal genes locus and contributes to its transcriptional silencing maintenance. Int. J. Biochem. Cell Biol. *110*, 143–148. https://doi.org/10.1016/j.biocel.2019.03.006.
17. Reeder, R.H., and Lang, W.H. (1997). Terminating transcription in eukaryotes: lessons learned from RNA polymerase I. Trends Biochem. Sci. *22*, 473–477. https://doi.org/10.1016/s0968-0004(97)01133-x.
18. Prescott, E.M., Osheim, Y.N., Jones, H.S., Alen, C.M., Roan, J.G., Reeder, R.H., Beyer, A.L., and Proudfoot, N.J. (2004). Transcriptional termination by RNA polymerase I requires the small subunit Rpa12p. Proc. Natl. Acad. Sci. USA *101*, 6068–6073. https://doi.org/10.1073/pnas.0401393101.
19. Lang, W.H., and Reeder, R.H. (1993). The REB1 site is an essential component of a terminator for RNA polymerase I in Saccharomyces cerevisiae. Mol. Cell Biol. *13*, 649–658. https://doi.org/10.1128/mcb.13.1.649-658.1993.
20. Lang, W.H., and Reeder, R.H. (1995). Transcription termination of RNA polymerase I due to a T-rich element interacting with Reb1p. Proc. Natl. Acad. Sci. USA *92*, 9781–9785. https://doi.org/10.1073/pnas.92.21.9781.
21. El Hage, A., Koper, M., Kufel, J., and Tollervey, D. (2008). Efficient termination of transcription by RNA polymerase I requires the 5' exonuclease Rat1 in yeast. Genes Dev. 22, 1069–1081.
22. Kawauchi, J., Mischo, H., Braglia, P., Rondon, A., and Proudfoot, N.J. (2008). Budding yeast RNA polymerases I and II employ parallel mechanisms of transcriptional termination. Genes Dev. *22*, 1082–1092. https://doi.org/10.1101/gad.463408.
23. Kufel, J., Dichtl, B., and Tollervey, D. (1999). Yeast Rnt1p is required for cleavage of the pre-ribosomal RNA in the 3' ETS but not the 5' ETS. RNA *5*, 909–917. https://doi.org/10.1017/s135583829999026x.
24. Ha, C.W., Sung, M.K., and Huh, W.K. (2012). Nsi1 plays a significant role in the silencing of ribosomal DNA in Saccharomyces cerevisiae. Nucleic Acids Res. *40*, 4892–4903. https://doi.org/10.1093/nar/gks188.
25. Reiter, A., Hamperl, S., Seitz, H., Merkl, P., Perez-Fernandez, J., Williams, L., Gerber, J., Németh, A., Léger, I., Gadal, O., et al. (2012). The Reb1-homologue Ydr026c/Nsi1 is required for efficient RNA polymerase I

termination in yeast. EMBO J. *31*, 3480–3493. https://doi.org/10.1038/emboj.2012.185.

26. Merkl, P., Perez-Fernandez, J., Pilsl, M., Reiter, A., Williams, L., Gerber, J., Böhm, M., Deutzmann, R., Griesenbeck, J., Milkereit, P., and Tschochner, H. (2014). Binding of the termination factor Nsi1 to its cognate DNA site is sufficient to terminate RNA polymerase I transcription in vitro and to induce termination in vivo. Mol. Cell Biol. *34*, 3817–3827. https://doi.org/10.1128/mcb.00395-14.

27. Clarke, A.M., Huffines, A.K., Edwards, Y.J.K., Petit, C.M., and Schneider, D.A. (2021). Defining the Influence of the A12.2 Subunit on Transcription Elongation and Termination by RNA Polymerase I In Vivo. Genes *12*, 1939. https://doi.org/10.3390/genes12121939.

28. Ford, B.L., Wei, T., Liu, H., Scull, C.E., Najmi, S.M., Pitts, S., Fan, W., Schneider, D.A., and Laiho, M. (2023). Expression of RNA polymerase I catalytic core is influenced by RPA12. PLoS One *18*, e0285660. https://doi.org/10.1371/journal.pone.0285660.

29. Kuhn, C.D., Geiger, S.R., Baumli, S., Gartmann, M., Gerber, J., Jennebach, S., Mielke, T., Tschochner, H., Beckmann, R., and Cramer, P. (2007). Functional Architecture of RNA Polymerase I. Cell *131*, 1260–1272. https://doi.org/10.1016/j.cell.2007.10.051.

30. Scull, C.E., Lucius, A.L., and Schneider, D.A. (2021). The N-terminal domain of the A12.2 subunit stimulates RNA polymerase I transcription elongation. Biophys. J. *120*, 1883–1893. https://doi.org/10.1016/j.bpj.2021.03.007.

31. Schwank, K., Schmid, C., Fremter, T., Milkereit, P., Griesenbeck, J., and Tschochner, H. (2022). RNA polymerase I (Pol I) lobe-binding subunit Rpa12.2 promotes RNA cleavage and proofreading. J. Biol. Chem. *298*, 101862. https://doi.org/10.1016/j.jbc.2022.101862.

32. Van Mullem, V., Landrieux, E., Vandenhaute, J., and Thuriaux, P. (2002). Rpa12p, a conserved RNA polymerase I subunit with two functional domains. Mol. Microbiol. *43*, 1105–1113. https://doi.org/10.1046/j.1365-2958.2002.02824.x.

33. Braglia, P., Kawauchi, J., and Proudfoot, N.J. (2011). Co-transcriptional RNA cleavage provides a failsafe termination mechanism for yeast RNA polymerase I. Nucleic Acids Res. *39*, 1439–1448. https://doi.org/10.1093/nar/gkq894.

34. Gromak, N., West, S., and Proudfoot, N.J. (2006). Pause sites promote transcriptional termination of mammalian RNA polymerase II. Mol. Cell Biol. *26*, 3986–3996. https://doi.org/10.1128/MCB.26.10.3986-3996.2006.

35. Lesne, A., Victor, J.-M., Bertrand, E., Basyuk, E., and Barbi, M. (2018). The Role of Supercoiling in the Motor Activity of RNA Polymerases. In Molecular Motors: Methods and Protocols, C. Lavelle, ed. (Springer), pp. 215–232. https://doi.org/10.1007/978-1-4939-8556-2_11.

36. Tantale, K., Mueller, F., Kozulic-Pirher, A., Lesne, A., Victor, J.-M., Robert, M.-C., Capozi, S., Chouaib, R., Bäcker, V., Mateos-Langerak, J., et al. (2016). A single-molecule view of transcription reveals convoys of RNA polymerases and multi-scale bursting. Nat. Commun. *7*, 12248. https://doi.org/10.1038/ncomms12248.

37. Heberling, T., Davis, L., Gedeon, J., Morgan, C., and Gedeon, T. (2016). A Mechanistic Model for Cooperative Behavior of Co-transcribing RNA Polymerases. PLoS Comput. Biol. *12*, e1005069. https://doi.org/10.1371/journal.pcbi.1005069.

38. Mendoza-Ochoa, G.I., Barrass, J.D., Terlouw, B.R., Maudlin, I.E., de Lucas, S., Sani, E., Aslanzadeh, V., Reid, J.A.E., and Beggs, J.D. (2019). A fast and tuneable auxin-inducible degron for depletion of target proteins in budding yeast. Yeast *36*, 75–81. https://doi.org/10.1002/yea.3362.

39. Fang, F., Phillips, S., and Butler, J.S. (2005). Rat1p and Rai1p function with the nuclear exosome in the processing and degradation of rRNA precursors. RNA *11*, 1571–1578. https://doi.org/10.1261/rna.2900205.

40. Xue, Y., Bai, X., Lee, I., Kallstrom, G., Ho, J., Brown, J., Stevens, A., and Johnson, A.W. (2000). Saccharomyces cerevisiae RAI1 (YGL246c) is homologous to human DOM3Z and encodes a protein that binds the nuclear exoribonuclease Rat1p. Mol. Cell Biol. *20*, 4006–4015. https://doi.org/10.1128/MCB.20.11.4006-4015.2000.

41. Kenna, M., Stevens, A., McCammon, M., and Douglas, M.G. (1993). An essential yeast gene with homology to the exonuclease-encoding *XRN1/KEM1* gene also encodes a protein with exoribonuclease activity. Mol. Cell Biol. *13*, 341–350. https://doi.org/10.1128/mcb.13.1.341-350.1993.

42. Delan-Forino, C., Schneider, C., and Tollervey, D. (2017). Transcriptome-wide analysis of alternative routes for RNA substrates into the exosome complex. PLoS Genet. *13*, e1006699. https://doi.org/10.1371/journal.pgen.1006699.

43. Neil, H., Malabat, C., d'Aubenton-Carafa, Y., Xu, Z., Steinmetz, L.M., and Jacquier, A. (2009). Widespread bidirectional promoters are the major source of cryptic transcripts in yeast. Nature *457*, 1038–1042. https://doi.org/10.1038/nature07747.

44. Tuck, A.C., and Tollervey, D. (2013). A transcriptome-wide atlas of RNP composition reveals diverse classes of mRNAs and lncRNAs. Cell *154*, 996–1009. https://doi.org/10.1016/j.cell.2013.07.047.

45. Liang, S., Hitomi, M., Hu, Y.H., Liu, Y., and Tartakoff, A.M. (1996). A DEAD-box-family protein is required for nucleocytoplasmic transport of yeast mRNA. Mol. Cell Biol. *16*, 5139–5146. https://doi.org/10.1128/MCB.16.9.5139.

46. de la Cruz, J., Kressler, D., Tollervey, D., and Linder, P. (1998). Dob1p (Mtr4p) is a putative ATP-dependent RNA helicase required for the 3' end formation of 5.8S rRNA in Saccharomyces cerevisiae. EMBO J. *17*, 1128–1140. https://doi.org/10.1093/emboj/17.4.1128.

47. Thoms, M., Thomson, E., Baßler, J., Gnädig, M., Griesel, S., and Hurt, E. (2015). The Exosome Is Recruited to RNA Substrates through Specific Adaptor Proteins. Cell *162*, 1029–1038. https://doi.org/10.1016/j.cell.2015.07.060.

48. Stevens, A., and Poole, T.L. (1995). 5'-exonuclease-2 of Saccharomyces cerevisiae. Purification and features of ribonuclease activity with comparison to 5'-exonuclease-1. J. Biol. Chem. *270*, 16063–16069. https://doi.org/10.1074/jbc.270.27.16063.

49. Petfalski, E., Dandekar, T., Henry, Y., and Tollervey, D. (1998). Processing of the precursors to small nucleolar RNAs and rRNAs requires common components. Mol. Cell Biol. *18*, 1181–1189. https://doi.org/10.1128/MCB.18.3.1181.

50. Amberg, D.C., Goldstein, A.L., and Cole, C.N. (1992). Isolation and characterization of RAT1: an essential gene of *Saccharomyces cerevisiae* required for the efficient nucleocytoplasmic trafficking of mRNA. Genes Dev. *6*, 1173–1189. https://doi.org/10.1101/gad.6.7.1173.

51. Cheung, A.C.M., and Cramer, P. (2011). Structural basis of RNA polymerase II backtracking, arrest and reactivation. Nature *471*, 249–253. https://doi.org/10.1038/nature09785.

52. Turowski, T.W., Leśniewska, E., Delan-Forino, C., Sayou, C., Boguta, M., and Tollervey, D. (2016). Global analysis of transcriptionally engaged yeast RNA polymerase III reveals extended tRNA transcripts. Genome Res. *26*, 933–944. https://doi.org/10.1101/gr.205492.116.

53. Delan-Forino, C., Spanos, C., Rappsilber, J., and Tollervey, D. (2020). Substrate Specificity of the TRAMP Nuclear Surveillance Complexes. Nat. Commun. *11*, 3122. https://doi.org/10.1038/s41467-020-16965-4.

54. Schneider, C., Kudla, G., Wlotzka, W., Tuck, A., and Tollervey, D. (2012). Transcriptome-wide analysis of exosome targets. Mol. Cell *48*, 422–433. https://doi.org/10.1016/j.molcel.2012.08.013.

55. Park, S.W., Parrott, A.M., Fritz, D.T., Park, Y., Mathews, M.B., and Lee, C.G. (2008). Regulation of the catalytic function of topoisomerase II alpha through association with RNA. Nucleic Acids Res. *36*, 6080–6090. https://doi.org/10.1093/nar/gkn614.

56. Peil, L., Waghmare, S., Fischer, L., Spitzer, M., Petfalski, E., Tollervey, D., and Rappsilber, J. (2018). Identification of RNA-associated peptides, iRAP, defines precise sites of protein-RNA interaction. Preprint at bioRxiv. https://doi.org/10.1101/456111. http://biorxiv.org/cgi/content/short/456111v1.

57. Winz, M.-L., Peil, L., Turowski, T.W., Rappsilber, J., and Tollervey, D. (2019). Molecular interactions between Hel2 and RNA supporting

CellPress
OPEN ACCESS

ribosome-associated quality control. Nat. Commun. *10*, 563. https://doi.org/10.1038/s41467-019-08382-z.

58. Wittner, M., Hamperl, S., Stöckl, U., Seufert, W., Tschochner, H., Milkereit, P., and Griesenbeck, J. (2011). Establishment and Maintenance of Alternative Chromatin States at a Multicopy Gene Locus. Cell *145*, 543–554. https://doi.org/10.1016/j.cell.2011.03.051.

59. Osheim, Y.N., French, S.L., Sikes, M.L., and Beyer, A.L. (2009). Electron Microscope Visualization of RNA Transcription and Processing in Saccharomyces cerevisiae by Miller Chromatin Spreading. Methods Mol. Biol. *464*, 55–69. https://doi.org/10.1007/978-1-60327-461-6_4.

60. Warner, J.R. (1999). The economics of ribosome biosynthesis in yeast. Trends Biochem. Sci. *24*, 437–440. https://doi.org/10.1016/s0968-0004(99)01460-7.

61. von der Haar, T. (2008). A quantitative estimation of the global translational activity in logarithmically growing yeast cells. BMC Syst. Biol. *2*, 87. https://doi.org/10.1186/1752-0509-2-87.

62. Ibars, E., Codina-Fabra, J., Bellí, G., Casas, C., Tarrés, M., Solé-Soler, R., Lorite, N.P., Ximénez-Embún, P., Muñoz, J., Colomina, N., and Torres-Rosell, J. (2023). Ubiquitin proteomics identifies RNA polymerase I as a target of the Smc5/6 complex. Cell Rep. *42*, 112463. https://doi.org/10.1016/j.celrep.2023.112463.

63. Richardson, L.A., Reed, B.J., Charette, J.M., Freed, E.F., Fredrickson, E.K., Locke, M.N., Baserga, S.J., and Gardner, R.G. (2012). A Conserved Deubiquitinating Enzyme Controls Cell Growth by Regulating RNA Polymerase I Stability. Cell Rep. *2*, 372–385. https://doi.org/10.1016/j.celrep.2012.07.009.

64. Oesterreich, F.C., Herzel, L., Straube, K., Hujer, K., Howard, J., and Neugebauer, K.M. (2016). Splicing of Nascent RNA Coincides with Intron Exit from RNA Polymerase II. Cell *165*, 372–381. https://doi.org/10.1016/j.cell.2016.02.045.

65. Milligan, L., Sayou, C., Tuck, A., Auchynnikava, T., Reid, J.E., Alexander, R., Alves, F.d.L., Allshire, R., Spanos, C., Rappsilber, J., et al. (2017). RNA polymerase II stalling at pre-mRNA splice sites is enforced by ubiquitination of the catalytic subunit. Elife *6*, e27082. https://doi.org/10.7554/eLife.27082.

66. Saldi, T., Cortazar, M.A., Sheridan, R.M., and Bentley, D.L. (2016). Coupling of RNA Polymerase II Transcription Elongation with Pre-mRNA Splicing. J. Mol. Biol. *428*, 2623–2635. https://doi.org/10.1016/j.jmb.2016.04.017.

67. Cortazar, M.A., Sheridan, R.M., Erickson, B., Fong, N., Glover-Cutter, K., Brannan, K., and Bentley, D.L. (2019). Control of RNA Pol II Speed by PNUTS-PP1 and Spt5 Dephosphorylation Facilitates Termination by a "Sitting Duck Torpedo" Mechanism. Mol. Cell *76*, 896–908.e4. https://doi.org/10.1016/j.molcel.2019.09.031.

68. Lisica, A., Engel, C., Jahnel, M., Roldán, É., Galburt, E.A., Cramer, P., and Grill, S.W. (2016). Mechanisms of backtrack recovery by RNA polymerases I and II. Proc. Natl. Acad. Sci. USA *113*, 2946–2951. https://doi.org/10.1073/pnas.1517011113.

69. El Hage, A., Webb, S., Kerr, A., and Tollervey, D. (2014). Genome-Wide Distribution of RNA-DNA Hybrids Identifies RNase H Targets in tRNA Genes, Retrotransposons and Mitochondria. PLoS Genet. *10*, e1004716. https://doi.org/10.1371/journal.pgen.1004716.

70. Farnung, L., Ochmann, M., Engeholm, M., and Cramer, P. (2021). Structural basis of nucleosome transcription mediated by Chd1 and FACT. Nat. Struct. Mol. Biol. *28*, 382–387. https://doi.org/10.1038/s41594-021-00578-6.

71. Schneider, D.A., French, S.L., Osheim, Y.N., Bailey, A.O., Vu, L., Dodd, J., Yates, J.R., Beyer, A.L., and Nomura, M. (2006). RNA polymerase II elongation factors Spt4p and Spt5p play roles in transcription elongation by RNA polymerase I and rRNA processing. Proc. Natl. Acad. Sci. USA *103*, 12707–12712. https://doi.org/10.1073/pnas.0605686103.

72. Longtine, M.S., McKenzie, A., 3rd, Demarini, D.J., Shah, N.G., Wach, A., Brachat, A., Philippsen, P., and Pringle, J.R. (1998). Additional modules for versatile and economical PCR-based gene deletion and modification in Saccharomyces cerevisiae. Yeast *14*, 953–961. https://doi.org/10.1002/(SICI)1097-0061(199807)14:10<953::AID-YEA293>3.0.CO;2-U.

73. Henry, Y., Wood, H., Morrissey, J.P., Petfalski, E., Kearsey, S., and Tollervey, D. (1994). The 5' end of yeast 5.8S rRNA is generated by exonucleases from an upstream cleavage site. EMBO J. *13*, 2452–2463. https://doi.org/10.1002/j.1460-2075.1994.tb06530.x.

74. Houseley, J., and Tollervey, D. (2006). Yeast Trf5p is a nuclear poly(A) polymerase. EMBO Rep. *7*, 205–211. https://doi.org/10.1038/sj.embor.7400612.

75. LaCava, J., Houseley, J., Saveanu, C., Petfalski, E., Thompson, E., Jacquier, A., and Tollervey, D. (2005). RNA degradation by the exosome is promoted by a nuclear polyadenylation complex. Cell *121*, 713–724. https://doi.org/10.1016/j.cell.2005.04.029.

76. Webb, S., Hector, R.D., Kudla, G., and Granneman, S. (2014). PAR-CLIP data indicate that Nrd1-Nab3-dependent transcription termination regulates expression of hundreds of protein coding genes in yeast. Genome Biol. *15*, R8. https://doi.org/10.1186/gb-2014-15-1-r8.

77. Li, H., Handsaker, B., Wysoker, A., Fennell, T., Ruan, J., Homer, N., Marth, G., Abecasis, G., and Durbin, R.; 1000 Genome Project Data Processing Subgroup (2009). The Sequence Alignment/Map format and SAMtools. Bioinformatics *25*, 2078–2079. https://doi.org/10.1093/bioinformatics/btp352.

78. Quinlan, A.R., and Hall, I.M. (2010). BEDTools: a flexible suite of utilities for comparing genomic features. Bioinformatics *26*, 841–842. https://doi.org/10.1093/bioinformatics/btq033.

79. Dobin, A., Davis, C.A., Schlesinger, F., Drenkow, J., Zaleski, C., Jha, S., Batut, P., Chaisson, M., and Gingeras, T.R. (2013). STAR: ultrafast universal RNA-seq aligner. Bioinformatics *29*, 15–21. https://doi.org/10.1093/bioinformatics/bts635.

80. Laughery, M.F., Hunter, T., Brown, A., Hoopes, J., Ostbye, T., Shumaker, T., and Wyrick, J.J. (2015). New vectors for simple and streamlined CRISPR-Cas9 genome editing in Saccharomyces cerevisiae. Yeast *32*, 711–720. https://doi.org/10.1002/yea.3098.

81. Granneman, S., Petfalski, E., and Tollervey, D. (2011). A cluster of ribosome synthesis factors regulate pre-rRNA folding and 5.8S rRNA maturation by the Rat1 exonuclease. EMBO J. *30*, 4006–4019. https://doi.org/10.1038/emboj.2011.256.

82. Dengl, S., and Cramer, P. (2009). Torpedo nuclease Rat1 is insufficient to terminate RNA polymerase II in vitro. J. Biol. Chem. *284*, 21270–21279. https://doi.org/10.1074/jbc.M109.013847.

83. Xiang, S., Cooper-Morgan, A., Jiao, X., Kiledjian, M., Manley, J.L., and Tong, L. (2009). Structure and function of the 5'->3' exoribonuclease Rat1 and its activating partner Rai1. Nature *458*, 784–788. https://doi.org/10.1038/nature07731.

84. Jiao, X., Chang, J.H., Kilic, T., Tong, L., and Kiledjian, M. (2013). A mammalian pre-mRNA 5' end capping quality control mechanism and an unexpected link of capping to pre-mRNA processing. Mol. Cell *50*, 104–115. https://doi.org/10.1016/j.molcel.2013.02.017.

85. Engel, C., Sainsbury, S., Cheung, A.C., Kostrewa, D., and Cramer, P. (2013). RNA polymerase I structure and transcription regulation. Nature *502*, 650–655. https://doi.org/10.1038/nature12712.

86. Dodt, M., Roehr, J.T., Ahmed, R., and Dieterich, C. (2012). FLEXBAR-Flexible Barcode and Adapter Processing for Next-Generation Sequencing Platforms. Biology *1*, 895–905. https://doi.org/10.3390/biology1030895.

87. Ramírez, F., Ryan, D.P., Grüning, B., Bhardwaj, V., Kilpert, F., Richter, A.S., Heyne, S., Dündar, F., and Manke, T. (2016). deepTools2: a next generation web server for deep-sequencing data analysis. Nucleic Acids Res. *44*, W160–W165. https://doi.org/10.1093/nar/gkw257.

## STAR★METHODS

### KEY RESOURCES TABLE

| REAGENT or RESOURCE | SOURCE | IDENTIFIER |
|---|---|---|
| Antibodies | | |
| TAP Tag Polyclonal Antibody | ThermoFisher | Cat#CAB1001 |
| Chemicals, peptides, and recombinant proteins | | |
| -Trp synthetic dropout mix | Formedium | Cat#DCS0149 |
| Guanidine hydrochloride | Sigma | Cat#G4505-1KG |
| HaloTEV Protease | Promega | Cat#G6601 |
| Critical commercial assays | | |
| cOmplete EDTA-free protease inhibitor cocktail tablets | Roche | Cat#11873580001 |
| Ni-NTA Superflow | QIAGEN | Cat#30410 |
| Calmodulin Affinity resin | Agilent | Cat#214303-52 |
| Pierce spin columns snap cap | Thermo Scientific | Cat#69725 |
| Slide-A-lyzer cassette 2000MWCO | Thermo Scientific | Cat#66205 |
| RNace-It Ribonuclease cocktail | Agilent | Cat#400720 |
| RNasin Ribonuclease Inhibitor | Promega | Cat#N21150 |
| Recombinant RNasin Ribonuclease Inhibitor | Promega | Cat#N2511 |
| DNase RQ1 | Promega | Cat#M6101 |
| T4 RNA Ligase 2, truncated K227Q | NEB | Cat#M0351 |
| T4 RNA Ligase 1 | NEB | Cat#M0204L |
| T4 PNK | NEB | Cat#M0201L |
| Nitrocellulose membranes | GE Healthcare | Cat#10 439 196 |
| MetaPhor agarose | Lonza | Cat#50180 |
| NuPAGE 4–12% polyacrylamide Bis-Tris Gels | Life Technologies | Cat#NP0335 |
| NuPAGE LDS 4x sample buffer | Life Technologies | Cat#NP0007 |
| NuPAGE SDS-MOPS running buffer | Life Technologies | Cat#NP0001 |
| NuPAGE Transfer Buffer | Life Technologies | Cat#NP00061 |
| MinElute Gel Extraction kit | QIAGEN | Cat#28604 |
| Proteinase K | Roche | Cat#03115836001 |
| RNase H | NEB | Cat#M0297L |
| LA Taq | Takara | Cat#RR002M |
| Deposited data | | |
| Raw data files from CRAC | NCBI Gene expression omnibus | GSE136056 |
| Raw image files | Mendeley | https://doi.org/10.17632/m253kk9sm6.1 |
| Experimental models: Organisms/strains | | |
| S. cerevisiae Strain background: BY4741 (MATa his3Δ1 leu2Δ0 met15Δ0 ura3Δ0) | Longtine et al.[72] | yTWT001 |
| S. cerevisiae Strain Rpa190HTP a his3Δ1 leu2Δ0 met15Δ0 ura3Δ0 RPA190-HTP::URA3MX | Turowski et al.[7] | yTWT046 |
| S. cerevisiae Strain Rpa135 HTP a his3Δ1 leu2Δ0 met15Δ0 ura3Δ0 RPA135-HTP::URA3MX | Turowski et al.[7] | yTWT051 |
| S. cerevisiae Strain Rpa135 HTP Rpa12ΔC a his3Δ1 leu2Δ0 met15Δ0 ura3Δ0 RPA12(1-74aa only) RPA135-HTP::URA3MX | This study | yTWT232 |
| S. cerevisiae Strain nsi1Δ Rpa135 HTP a his3Δ1 leu2Δ0 met15Δ0 ura3Δ0 RPA135-HTP::URA3MX nsi1ΔHIS | This study | D1436 |

(*Continued on next page*)

***Continued***

| REAGENT or RESOURCE | SOURCE | IDENTIFIER |
|---|---|---|
| S. cerevisiae Strain nsi1Δ Rpa135 HTP Rpa12ΔC a his3Δ1 leu2Δ0 met15Δ0 ura3Δ0 RPA12(1-74aa only) RPA135-HTP::URA3MX nsi1ΔHIS | This study | D1433 |
| S.cerevisiae strain Rat1-TAP::HIS a his3Δ1 leu2Δ0 met15Δ0 ura3Δ0 Rat1-TAP::HIS | Open biosystems | N/A |
| S.cerevisiae strain Rai1-TAP::URA a his3Δ1 leu2Δ0 met15Δ0 ura3Δ0 Rai1-TAP::URA | This study | D1375 |
| S cerevisiae strain Trf4-TAP a his3D1 leu2D0 met15D0 ura3D0 TRF4-TAP-his | Open biosystems | N/A |
| S cerevisiae strain rat1-1 ura3-52, leu2Δ1,his3-200 rat1-1 | Henry et al.[73] | DAH18 |
| S cerevisiae strain rat1-1 ura3-52, leu2Δ1,his3-200 rat1-1, Rpa190-HTP | This study | D1464 |
| S cerevisiae strain trf4ts-gfp-kan trf5Δnat his3Δ1 leu2Δ0 met15Δ0 ura3Δ0 | Houseley and Tollervey[74] | JH385 |
| S cerevisiae strain trf4ts-gfp-kan trf5Δnat his3Δ1 leu2Δ0 met15Δ0 ura3Δ0 Rpa190-HTP | This study | D1457 |
| S cerevisiae strain air1Δnat, air2Δkan MATa his3Δ1 leu2Δ0 met15Δ0 ura3Δ0 air1Δ:nat air2Δ:kanMATa | LaCava et al.[75] | JH696 |
| S cerevisiae strain air1Δnat, air2Δkan MATa his3Δ1 leu2Δ0 met15Δ0 ura3Δ0 air1Δ:nat air2Δ:kanMATa Rpa190-HTP | This study | D1474 |
| S cerevisiae strain trf4ts-gfp-kan trf5Δnat his3Δ1 leu2Δ0 met15Δ0 ura3Δ0 Rpa190-HTP | This study | D1457 |
| S cerevisiae strain AID-RNT1 RPA190-HTP a leu2Δ0 ura3Δ0 his3Δ1:OsTIR:NatMX HygMX:pMET::AID-6xFLAG-RNT1 RPA190-HTP::URA3MX | This study | yTWT195 |
| S cerevisiae strain A190-HTP Rrp44-D551N a his3Δ1 leu2Δ0 met15Δ0 ura3Δ0 RPA190-HTP::URA3MX, Rrp44 D551N | This study | yTWT298 |
| S cerevisiae strain Rpa135-HTP Rpa12ΔC Rrp44-D551N a his3Δ1 leu2Δ0 met15Δ0 ura3Δ0 RPA12(1-74aa only) RPA135-HTP::URA3MX, Rrp44 D551N | This study | yTWT300 |
| S cerevisiae strain A135-HTP Rrp44-D551N a his3Δ1 leu2Δ0 met15Δ0 ura3Δ0 RPA135-HTP::URA3MX Rrp44 D551N | This study | yTWT302 |
| Software and algorithms | | |
| PyCRAC | Webb et al.[76] | https://bitbucket.org/sgrann/pycrac |
| SAMtools v1.3.1 | Li et al.[77] | http://www.htslib.org/ |
| Bedtools v2.25 | Quinlan and Hall[78] | https://github.com/arq5x/bedtools2 |
| Prism 7 | Graphpad | http://www.graphpad.com |
| Integrative Genomics Viewer | Broad Institute | http://software.broadinstitute.org/software/igv/ |
| STAR v2.7.10a | Dobin et al.[79] | N/A |
| trxtools 0.2.1 | This study | https://github.com/TurowskiLab/trxtools |
| MATLAB code for mathematical model: git repository | This study | https://github.com/tturowski/RNAPI-model-v2 https://doi.org/10.5281/zenodo.14587255 |
| Analysis steps code for mathematical model: git repository | This study | https://github.com/tturowski/Pol1_termination_MS https://doi.org/10.5281/zenodo.14592083 |

## EXPERIMENTAL MODEL AND STUDY PARTICIPANT DETAILS

### Strains

Yeast analyses were performed in strains derived from BY4741 (*MATa*; *his3Δ1*; *leu2Δ0*; *met15Δ0*; *ura3Δ0*). Rnt1 degron strain was constructed as described previously.[38] Rrp44 D551N scarless mutations were introduced using yeast CRISPR system.[80] For CRAC analyses, cells were grown in synthetic medium with 2% glucose at 30°C. Oligonucleotides used for strain construction are listed in Table S1.

## METHOD DETAILS

### *In-vivo* RNA crosslinking

Strains for CRAC experiments were grown in SD medium with 2% glucose, lacking tryptophan to $OD_{600}$ = 0.5. Actively growing cells were cross-linked in culture media using megatron UVC cross-linker[81] for 100 s or the Vari-X cross-linker for 8 s. Cells were spun and washed with 1x PBS buffer before freezing at −80°C.

### CRAC

Samples were processed as previously described.[52,57] However, phosphatase treatment was omitted, so the 3′-OH ends required for linker ligation are present only on nascent RNA transcripts. Cells were lysed in TNC100 (50mM Tris-HCl pH7.5, 150mM NaCl, 0.1% NP-40, 10mM $CaCl_2$, 5mM β-mercaptoethanol, 50U of DNase RQ1 and a protease-inhibitor cocktail (1 tablet/50mL) with zirconia beads in a 50mL conical. The cells were lysed with five 1-min pulses, with cooling on ice in between. The supernatant was spun for 20 min at 21,000g. The cleared lysate was incubated with the IgG Sepharose for 2 h at 4°C, with nutating. Subsequently, the beads were washed three times with TN1000 (50mM Tris-HCl pH7.5, 1000mM NaCl, 0.1% NP-40) and two times TN100 (50mM Tris-HCl pH7.5, 100mM NaCl, 0.1% NP-40). Protein:RNA complexes were eluted by incubation with HaloTEV for 2h at 18°C with shaking. The eluate was transferred to a fresh tube, 2.5U of RNace-IT was added and samples were incubated for 5 min at 37°C to fragment protein-bound RNA.

The 500 μL eluate was adjusted for nickel affinity purification with the addition of 400 mg guanidine hydrochloride, 45μL NaCl (3M) and 3μL imidazole (2.5M) and added to 100μL of washed nickel beads.

Following overnight incubation, the nickel beads were washed three times with WBI (6.0M guanidine hydrochloride, 50mM Tris-HCl pH7.5, 300mM NaCl, 0.1% NP-40, 10mM imidazole and 5mM β-mercaptoethanol), three times with PNK buffer (50mM Tris-HCl pH7.5, 50 mM NaCl, 1.5mM MgCl2, 0.1% NP-40, and 5mM β-mercaptoethanol) and transferred to a spin column. Subsequent reactions (80μL total volume for each) were performed in the columns, and afterward washed once with WBI and three times with PNK buffer:

1. 3′-linker ligation (1x PNK buffer (NEB), 10% PEG8000, 20U T4 RNA Ligase 2 truncated K227Q, 80U RNasIN, 80pmol pre-adenylated 3′ miRCat-33 linker (IDT); 24°C 6hrs).
2. 5′-end phosphorylation and radiolabeling (1x PNK buffer (NEB), 40U T4 PNK (NEB), 40μCi $^{32}$P-γATP; 37°C for 60min, with addition of 100nmol of ATP after 45min).
3. 5′-linker ligation (1x PNK buffer (NEB), 10% PEG8000, 40U T4 RNA ligase 1 (NEB), 80U RNasIN, linker, 200pmol 5′-linker, 1mM ATP; 16°C, overnight).

The beads were washed once with WBI and three times WBII (50mM Tris-HCl pH7.5, 50mM NaCl, 0.1% NP-40, 200mM imidazole, and 5mM β-mercaptoethanol) buffer. Protein:RNA complexes were eluted in 400μL of elution buffer (50mM Tris-HCl pH7.5, 50mM NaCl, 0.1% NP-40, 200mM imidazole, and 5mM β-mercaptoethanol) and TCA precipitated for 1h on ice. RNPs were pelleted at 21,000g for 20min, washed in cold acetone and resuspended in 30μL 1X NuPAGE sample loading buffer supplemented with 8% β-mercaptoethanol. The sample was denatured by incubation at 65°C for 5min, and run on a 4%–12% Bis-tris NuPAGE gel at 130V. The protein:RNA complexes were transferred to Hybond-C nitrocellulose membranes with NuPAGE MOPS transfer buffer for 2h at 100V.

Labeled RNA was detected by autoradiography. The appropriate region was excised from the membrane and treated with 0.2 μg/μL Proteinase K (50mM Tris-HCl pH7.5, 50mM NaCl, 0.5%SDS, 1mM EDTA; 2hr 55°C with shaking) in a 400μL reaction. The RNA component was isolated with a standard phenol:chloroform extraction followed by ethanol precipitation with 1μL of GlycoBlue. The RNA was reverse transcribed using Superscript III and the miRCat-33 RT oligo (IDT) for 1h at 50°C in a 20μL reaction. The resulting cDNA was amplified by PCR in 50μL reactions using La Taq (5 μL template, 21–26 cycles) PCR reactions were combined, precipitated in ethanol, and resolved on a 3%Metaphore agarose gel. A region corresponding to 140 to 200 bp was excised from the gel and extracted using the Min-elute kit. Libraries were measured with Qbit and sequenced using Illumina HiSeq or llumina MiniSeq with 75bp single-end reads.

### Tap purification of Rat1, Rai1 and Trf4

We attempted to co-express tagged Rat1 and Rai1 in *E.coli* and purified the complex,[40,82–84] but due to low activity of the exonuclease we used Rat1-Rai1 complex purified from yeast. Strains were grown in YPD to OD 1.5, harvested, washed with ice-cold PBS and frozen at −80°C. Lysis was performed in TMN100 buffer as previously described. Lysates were bound to IgG-sepharose

for 1.5 h, washed 3 times in TMN100 and eluted with Halo-TEV at 24°C for 2 h. TEV eluate was bound to Calmodulin resin for 2 h at 4°C, washed 3 times in TMN150/2 mM CaCl2 and eluted with 5mM EGTA, 10 mM Tris pH 8, 50 mM NaCl. 500µL of eluate were injected into a Slide-A-lyzer cassette and dialyzed for 3h against TMN75/50% glycerol.

## Purification of RNA polymerase I and *in vitro* assay

Strains were grown in YPD to $OD_{600}$ = 1.5–2. Cells were span and washed with 1x PBS buffer before freezing at −80°C. Cells were lysed in Lysis buffer (50mM HEPES pH7.8, 400mM $(NH_4)_2SO_4$,10% glycerol, 40mM $MgCl_2$, 3mM DTT, 10mM $CaCl_2$, 50U of DNase RQ1 and a protease-inhibitor cocktail (1 tablet/50mL) with zirconia beads in a 50mL conical. The cells were lysed with ten 1-min pulses, with cooling on ice in between. The supernatant was spun for 20 min at 21,000g.

The protein content of the supernatant was determined using the Bradford assay. Equal protein amounts (usually 1mL cell extract, 20–30 mg) were incubated with 50–75µL of immunoglobulin-G (rabbit IgG, I5006, Sigma) coupled magnetic beads slurry (Dynabeads M-270 Epoxy, 300mg) for 1–2 h on a rotating wheel. The beads had previously been equilibrated with lysis buffer. The beads were washed four times with 1 mL buffer B1500 (20mM HEPES/KOH pH7.8, 1.5 M KOAc, 1mM $MgCl_2$, 10% glycerol, 0.1% IGEPAL CA-630) and three times with 1mL buffer B200 (20mM HEPES/KOH pH7.8, 200mM KOAc, 1mM $MgCl_2$, 10% glycerol). For elution, beads were finally resuspended in 400µL of TMN150 buffer (50mM Tris pH7.8, 150mM NaCl, 1.5mM $MgCl_2$, 0.1% NP40, 5mM βMeEth), supplemented with 3µL TEV protease (HaloTEV, Promega G6602) and incubated for 2h at 24°C in a thermomixer (1,000 rpm). The supernatant was collected, glycerol was added to 10% and aliquots were stored at −20°C for short term or at −80°C for longer. For buffer exchange assays, TEV elution was omitted, and aliquots were stored only for short term at 4°C. 10% of the purified fraction was analyzed via SDS–PAGE to monitor the purification success. Protein concentrations were determined by comparing the intensity of Coomassie-stained RNA polymerase subunits to the defined amount of Coomassie-stained HaloTEV protease used.

The *in vitro* RNA extension assay was modified from.[29,85] For 1 reaction, 2pmol of annealed RNA-DNA-DNA scaffold was preincubated with ∼2pmol of purified enzyme for 20 min at 20°C. Transcription was started by adding 6µL- 2x transcription buffer (TB). Elongation was performed in 1x TB (60mM $(NH_4)_2SO_4$, 20mM HEPES/KOH pH 7.6, 8mM $MgSO_4$, 10µM $ZnCl_2$, 10% glycerol, 10mM DTT) supplemented with 1mM NTPs. The samples were incubated at 28°C for 5min. For backtracking assays, reaction tubes were placed on a magnetic rack, and supernatant was removed. Beads were washed with 200µL buffer B200, resuspended in 12µL 1x TB without NTPs and incubated at 28°C for 10min. All reactions were stopped by addition of 2x RNA loading dye (Thermo, R0641). Samples were heat denatured at 80°C for 5min and resolved on 8M urea 20% polyacrylamide gels. Fluorescently labeled transcripts were visualized using a FujiFilm FLA-5100 Fluorescent Image Analyser. Images were processed using Multi Gauge software (Fuji). Oligonucleotides used for *in vitro* assays are listed in Table S2.

## Polyadenylation assays

For 1 reaction, 2pmol of annealed RNA-biotinDNA-DNA scaffold bound to Streptavidin magnetic beads was preincubated with ∼2pmol of purified enzyme for 20 min at 20°C. Beads were washed twice with B200 buffer. Transcription was started by adding 6µL 2x transcription buffer (TB). Elongation was performed in 1xTB (60mM $(NH_4)_2SO_4$, 20mM HEPES/KOH pH 7.6, 8 mM $MgSO_4$, 10µM $ZnCl_2$, 10mM DTT) supplemented with 1mM NTPs-GTP containing 0.5µL [α-$^{32}$P]-ATP at 28°C for 10 min. Beads were washed 3 times with B200 and resuspended in 1xTB. ∼2pmol of purified Trf4,1mM ATP were added and timepoints were taken at points 0, 5, 15 and 30 min. Poly(A)-tails were analyzed on 15% Acrylamide/Urea gels.

## Termination assays

For the scaffold of the termination assays 5′ kinased input RNA was used.

For 1 reaction, 2pmol of annealed RNA-DNA-DNAbiotin scaffold bound to Streptavidin magnetic beads was preincubated with ∼2pmol of purified enzyme for 20 min at 20°C. Beads were washed twice with B200 buffer. Transcription was started by adding 6µL 2x transcription buffer (TB). Elongation was performed in 1x TB (60mM $(NH_4)_2SO_4$, 20mM HEPES/KOH pH 7.6, 8mM $MgSO_4$, 10µM $ZnCl_2$, 10mM DTT) supplemented with 1mM NTPs-GTP containing 0.5µL [α-$^{32}$P]-ATP at 28C for 10 min. Beads were washed 3 times with B200 and resuspended in 1xTB. ∼2pmol of purified Rat1/Rai1 were added and timepoints were taken at point 0, 5, 15,30 and 60 min. PolyA-tails were analyzed on 15% Acrylamide/Urea gels.

To follow the protein drop-off rate, the termination assay was performed omitting the [α-$^{32}$P]-ATP. Samples were analyzed on precast SDS 4–12% gradient gels and westerns were done with TAP-antibody. Drop off rate was calculated using ImageJ.

## Modification of mathematical model for RNAPI transcription

The numerical model to estimate premature termination was developed on the basis of previously developed model[7] as described in the main text.

### *Simulation conditions*

Most of the simulation conditions was used as previously: gene length 7000 [nt], RNAPI size 38 [nt], RNA-DNA hybrid within the transcription bubble 11 [nt], transcription initiation probability 0.8, average RNAPI velocity 50 [nt·s$^{-1}$]. Model parameters: $dG^{Structure}_{Strength}$ 1.25, $dG^{RNA:DNA}_{Strength}$ which was represented as a ratio to $dG^{Structure}_{Strength}$, with ratio = 0.48, structure2consider = −11. Time step was 0.004 [s]. To simulate a full division cycle, we extended simulation time for 6000 [s] (=100 min) and run parallel simulations ($n$ = 16). To calculate output of 75 transcription units (∼50% active out of 150) we multiplied the output by 4.7.

Given alternative models we tested following parameter combinations: DNA stiffness constant c = {0.500},: $dG^{Structure}_{Strength}$ = {0,1.25}, premature termination probability = {0, 0.01, 0.02, 0.03, 0.04, 0.05, 0.06, 0.07, 0.08, 0.09, 0.1, 0.2, 0.3, 0.4, 0.5, 0.6, 0.7, 0.8, 0.9, 1}i, premature termination distance = {2000, 6750}. This gives us a total of 226 sets of parameters which were subsequently evaluated to ensure that the chosen parameter set gives a representative data, approximately in the middle of the set of simulations with neighboring parameters.

Full set of parameters was uploaded to the repository as CSV file runParameters.csv https://github.com/tturowski/RNAPI-model-v2. Output data were saved in MAT format and analyzed using jupyter-notebook uploaded to https://github.com/tturowski/Pol1_termination_MS as Fig_6_model_output.ipynb.

## QUANTIFICATION AND STATISTICAL ANALYSIS

### Analysis reproducibility

To support transparency and reproducibility of the analysis all steps were performed using snakemake pipeline and jupyter-notebooks. The files were deposited in open git repository https://github.com/tturowski/Pol1_termination_MS.

### Pre-processing and data alignment

Illumina sequencing data were demultiplexed using in-line barcodes and in this form were submitted to GEO. First quality control step was performed using FastQC software (http://www.bioinformatics.babraham.ac.uk/projects/fastqc/) considering specificity of CRAC data.

All processing steps were performed using snakemake pipeline (v7.14.0 [34035898]). Raw reads were collapsed to remove PCR duplicates using FASTX-collapser v0.0.14 (http://hannonlab.cshl.edu/fastx_toolkit/) then inline barcodes were removed using FASTX-trimmer v0.0.14. The 3′-adapters were removed using flexbar v3.5.0[86] with parameters -ao 4 –u 3 -m 7 -n 4 -bt RIGHT, and filtered to retain only reads containing the 3′-adapter.

All datasets were aligned to the yeast genome (sacCer3, EF4.74) using STAR v2.7.10a and saved as bam file format. Bam files were sorted and indexed using samtools v1.15.1.[77]

Second quality control step was performed using featureCounts v2.0.1 which calculates overlaps between aligned cDNAs and yeast genomic features. BigWig files were generated using bamCoverage script from deepTools package v3.5.1.[87]

### Selection of the 5′-ends, 3′-ends and polyA

To prepare BigWig files containing the 5′-ends of reads, or the 3′-ends of reads, or the 3′-ends of poly-adenylated reads (AAA or longer) custom tool SAM2profilesGenomic.py from trxtools v0.2.2 was used. The package is publicly available https://pypi.org/project/trxtools/0.2.2/.

### RNA polymerase I profile

Downstream analyses were performed using python 2.7 Jupyter notebooks, python libraries (pandas v0.19.2, numpy v1.16.0, scipy v1.2.0, matplotlib v2.2.3) and in-house functions available in trxtools, created as modification of gwide toolkit published previously.[52] All reads mapping to the gene encoding pre-rRNA (*RDN37* gene with 1300 nt overhangs) were summed up to 1 and fraction of reads was used further, adding $10^{-7}$ pseudo count. There are two copies of the *RDN37* gene in the reference genome; *RDN37-1* and *RDN37-2.* Subsequent analyses used the combination of *RDN37-1* and *RDN37*-2 gene or *RDN37-2* for analysis of transcriptional read-trough.

To smooth data we used centered Blackman function (window 10). CRAC profiles were presented similar to boxplots of six biological replicates: median as a solid line, range between second and third quartile with darker color and range between minimum and maximum as lighter color. The basic profile of RNAPI CRAC was established as previously described.[7]

### Peak calling and metaplots

Peak calling was performed using findPeaks function from trxtools v0.2.2 using order value 45 and window 80. To generate peak metaplot for each peak or trough, a two sided window around the feature was superimposed with all other peaks. Mean for all windows were calculated and data for each dataset were presented as peak metaplot. Metaplots were generated using cumulativePeaks function from trxtools v0.2.2.

### Statistical analyses and numerical methods

Most of the plots and statistical analyses of this work were performed using python 3.6, Jupyter notebooks and python library scipy v1.2.0. Boxplots present 2$^{nd}$ and 3$^{rd}$ quartile, line marks median and whiskers range between 5$^{th}$ and 95$^{th}$ percentile. To visualize reproducible differences between strains we marked area between outside q2-q3 range.

The Western blot quantification was conducted using ImageJ software. To determine the RNAPI drop-off rate, the percentage change between the 0-min time point and the given time point was calculated as a ratio and then 100% was subtracted. This adjustment normalizes the 0-min time point to 0%, and the result represents the percentage of drop-off for subsequent time points.

Statistical significance was assessed using Wilcoxon signed-rank and rank sum tests, with the T test applied where appropriate (indicated in figure legend). All statistical tests were two-tailed, and the legend for *p*-values was consistently represented as follows: $^*p < 0.05$, $^{**}p < 0.01$, $^{***}p < 0.001$.

## ADDITIONAL RESOURCES

Any additional information required to reanalyze the data reported in this paper is available from the lead contact upon request.

**Cell Reports, Volume *44***

## Supplemental information

# Multiple mechanisms of termination modulate the dynamics of RNAPI transcription

**Elisabeth Petfalski, Marie-Luise Winz, Katarzyna Grelewska-Nowotko, Tomasz W. Turowski, and David Tollervey**

**SUPPLEMENTARY MATERIAL**


**Figure S1.** Distribution of Rpa190 along the rDNA (related to Figure 1)
**Figure S2.** RNAPI pause sites in transcription termination region (related to Figure 2)
**Figure S3.** Schematic of *in vitro* termination assay (related to Figure 3)
**Figure S4.** Rat1 and poly(A) tails are found at sites of slowed RNAPI elongation (related to Figure 4)
**Figure S5.** RNAPI and Top2 distributions along the 5' ETS (related to Figure 5)
**Figure S6.** Development of premature termination $P_{PT}$ function (related to Figure 6)

**Table S1.** Oligonucleotides used for strain construction
**Table S2.** Oligonucleotides used for *in vitro* assay


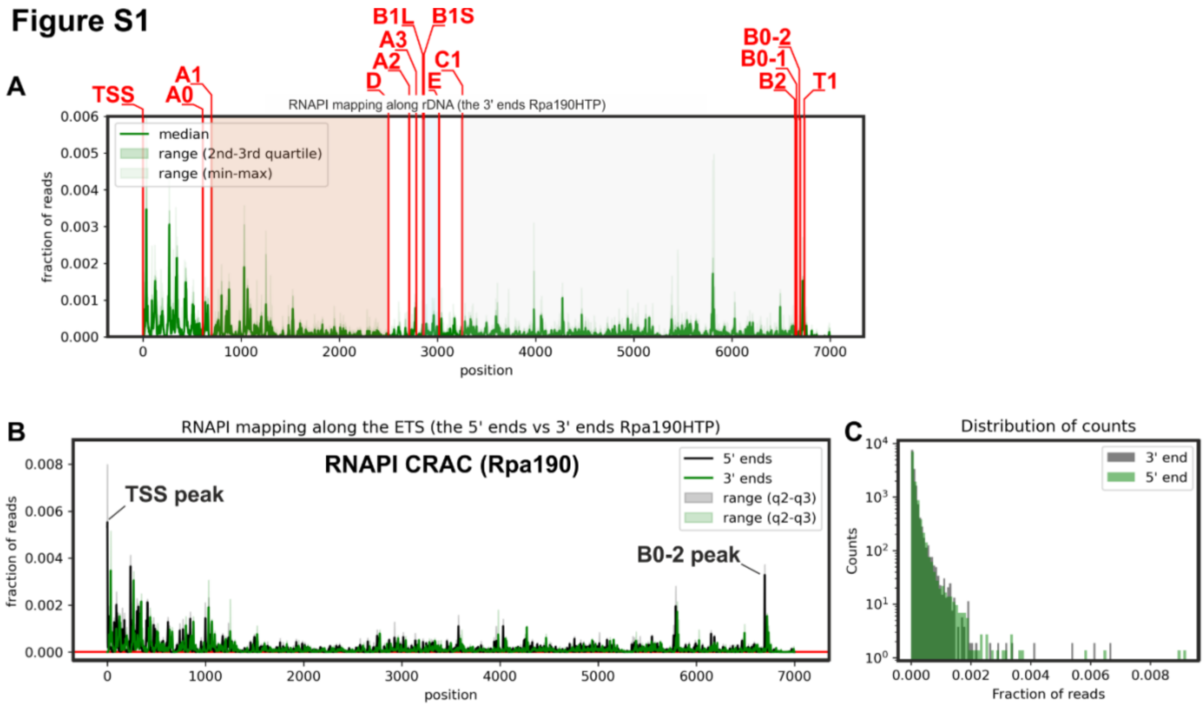


**Figure S1.** Distribution of Rpa190 along the rDNA (related to Figure 1). Data are represented as median (n=6).
A: Rpa190 distribution over rDNA transcription unit with marked processing sites.
B: RNAPI Rpa190-HTP CRAC mapping of the 5' ends (black) and the 3' ends (green) of reads. Note that substantial differences between the 5' and 3' ends are visible for the very 5' end at the transcription start site (TSS peak) and within the 3' ETS, at the B0-2 cleavage site (B0-2 peak).
C: 5' ends of RNAPI CRAC reads are less distributed relative to 3' ends.

# Figure S2

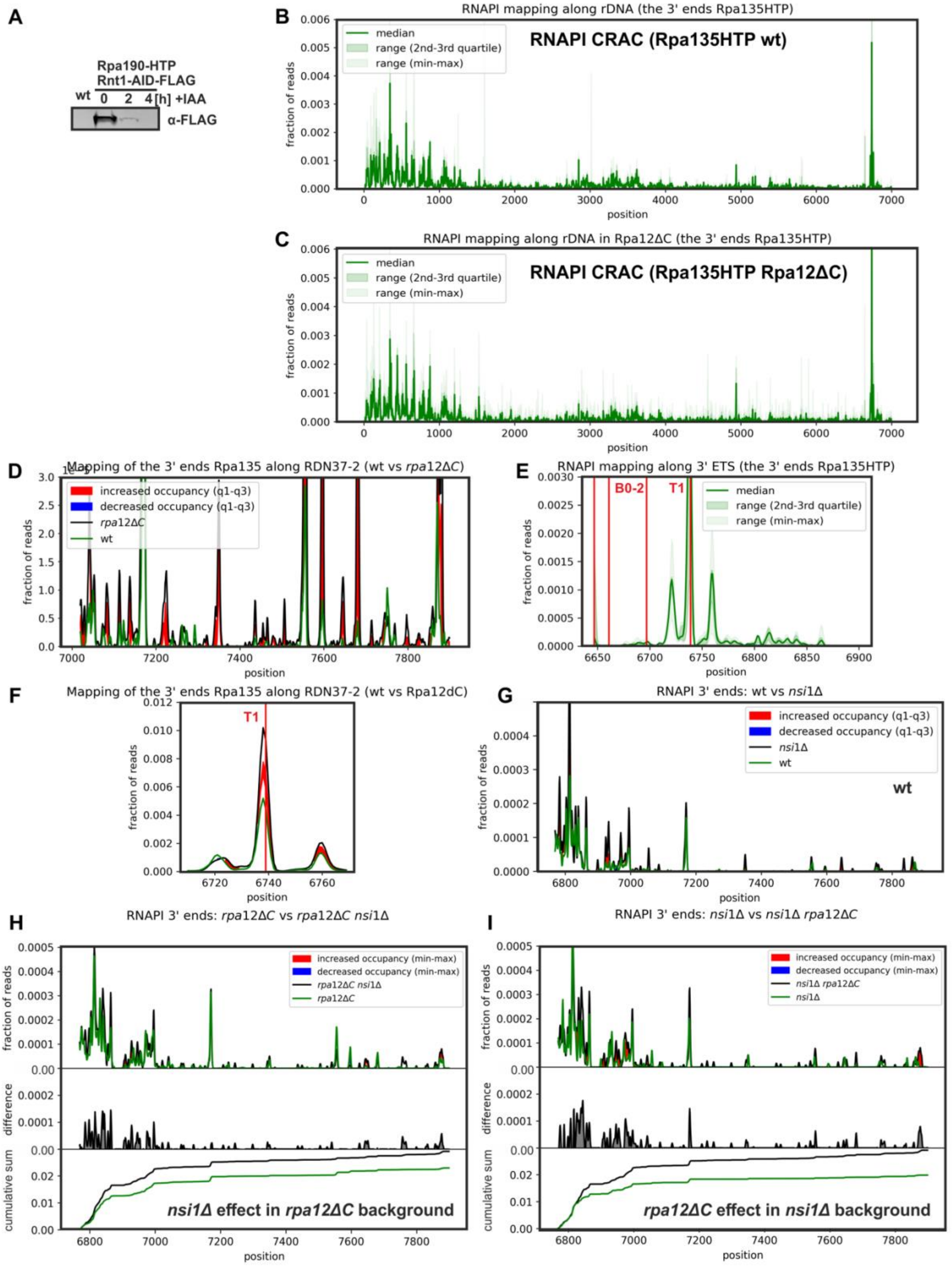


**Figure S2.** RNAPI pause sites in transcription termination region (related to Figure 2). Data are represented as median (Rpa135-HTP n=7, Rpa135-HTP *rpa12ΔC* n=4) or mean (Rpa135-HTP *nsi1ΔC* n=2, Rpa135-HTP *rpa12ΔC nsi1ΔC* n=2).

A: Depletion of Rnt1-AID-FLAG following indole acetic acid (IAA) addition to medium.

B: Distribution of RNAPI (Rpa135-HTP) CRAC 3’ ends of reads along the rDNA in the WT

C: Distribution of RNAPI (Rpa135-HTP) CRAC 3’ ends of reads along the rDNA in the *rpa12ΔC* strain
D: Distribution of RNAPI (Rpa135-HTP) downstream from the major T1 termination sites, comparing WT and *rpa12ΔC* strains. Differences are highlighted in color
E: Distribution of RNAPI Rpa135-HTP CRAC reads around the 3’ end of the 35S pre-rRNA.
F: RNAPI (Rpa135-HTP) CRAC signal is increased at the T2 site in the *rpa12ΔC* strain
G: Limited effect of *nsi1Δ* on RNAPI transcriptional read-through detected with Rpa135-HTP CRAC in the WT
H: Limited effect of *nsi1Δ* on RNAPI (Rpa135-HTP) transcriptional read-through in the *rpa12ΔC* background
I: Effect of *rpa12ΔC* on RNAPI (Rpa135-HTP) transcriptional read-trough in the *nsi1Δ* background

## Figure S3

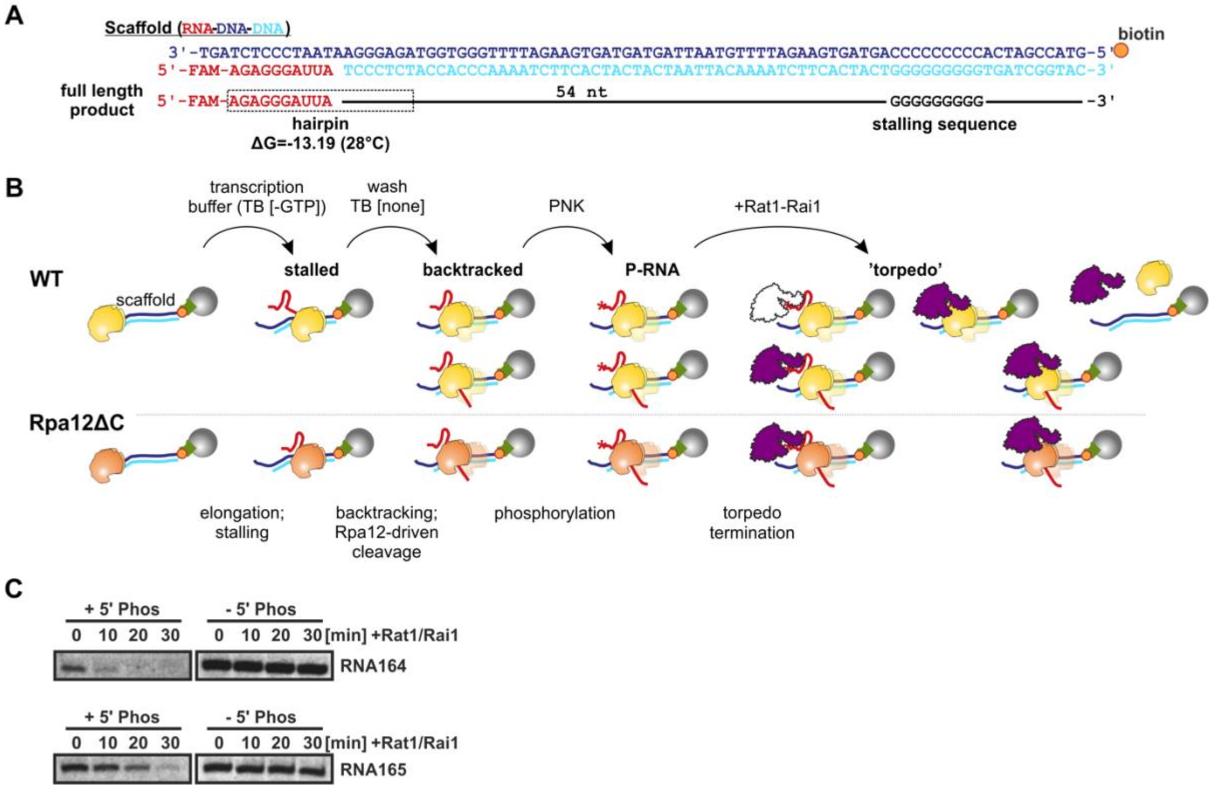


**Figure S3.** Schematic of *in vitro* termination assay (related to Figure 3).
A: Sequence of RNA-DNA-DNA scaffold immobilized via biotin-streptavidin interaction.
B: Schematic of the experiment.
C: Degradation of RNA oligonucleotides over a time course following addition of the purified Rat1-Rai1 complex, showing substantial protection in the absence of the 5’ monophosphate. Buffer and conditions as for termination assay (Fig. 3), but without addition of NTPs.

# Figure S4

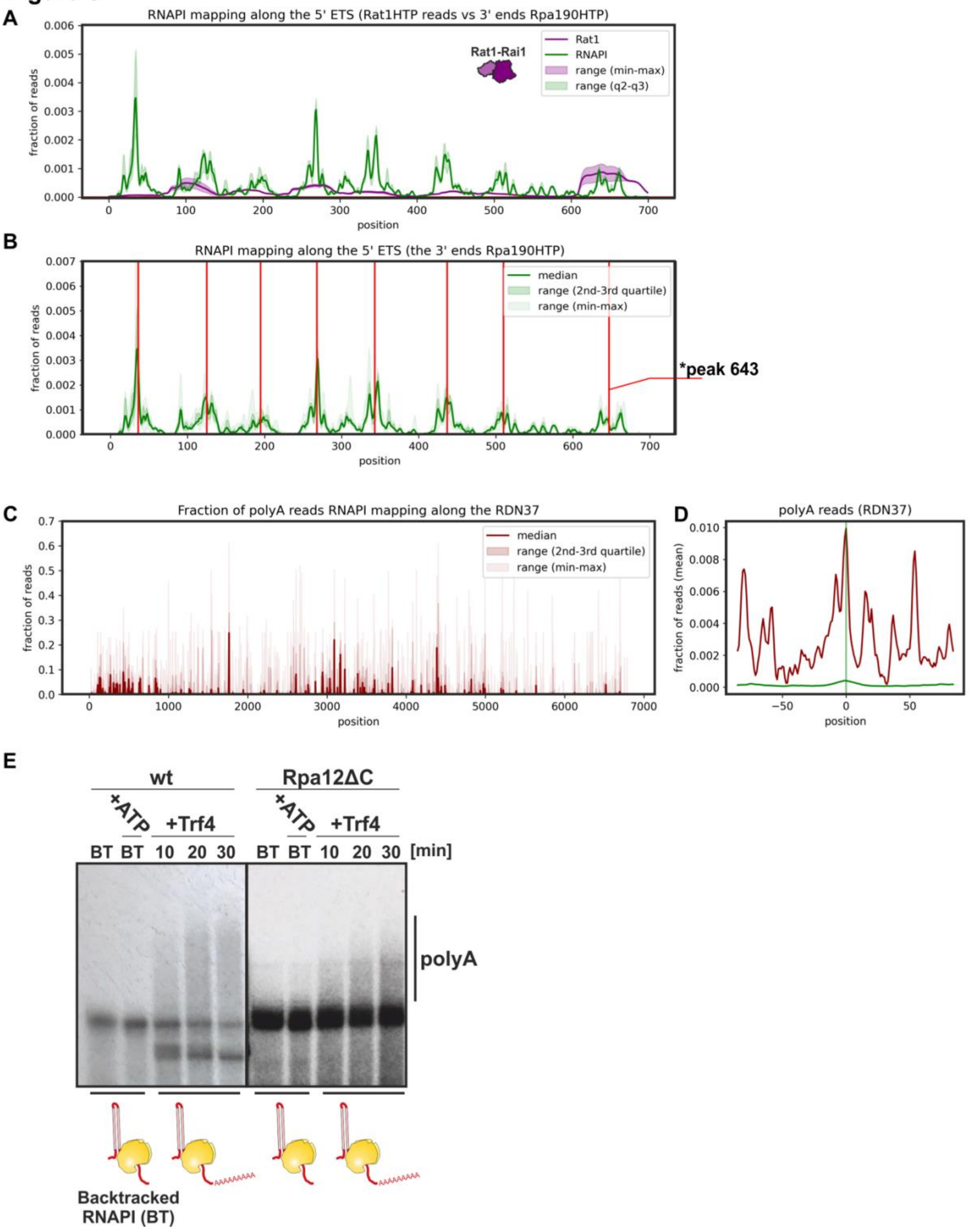


**Figure S4.** Rat1 and poly(A) tails are found at sites of slowed RNAPI elongation (related to Figure 4).
A: Rat1 total CRAC reads superimposed with the 3' ends of RNAPI CRAC reads. Data are represented as mean (n=2).
B: Peaks found with peak-calling algorithm that were used to generate peak metaplot. * Note peak 643 was excluded from meta-analysis to separate Rat1 surveillance from canonical degradation of the 5' ETS during pre-rRNA processing.
C: Fraction of poly(A) reads mapping along the rDNA (*RDN37*). Data are represented as mean (n=6).

D: RNAPI peak metaplot for *RDN37*, comparing the 3' ends of total Rpa190 reads (green) with poly(A) reads (red). As expected, the Rpa190 distribution matches the metapeak plot giving a low signal.

E: Trf4 oligo-adenylates the 3' end of backtracked, nascent RNA *in vitro* extruded from RNAPI. Comparison of adenylation on nascent transcripts associated with WT RNAPI (left) or RNAPI incorporating catalytically inactive Rpa12∆C (right).

**Figure S5**

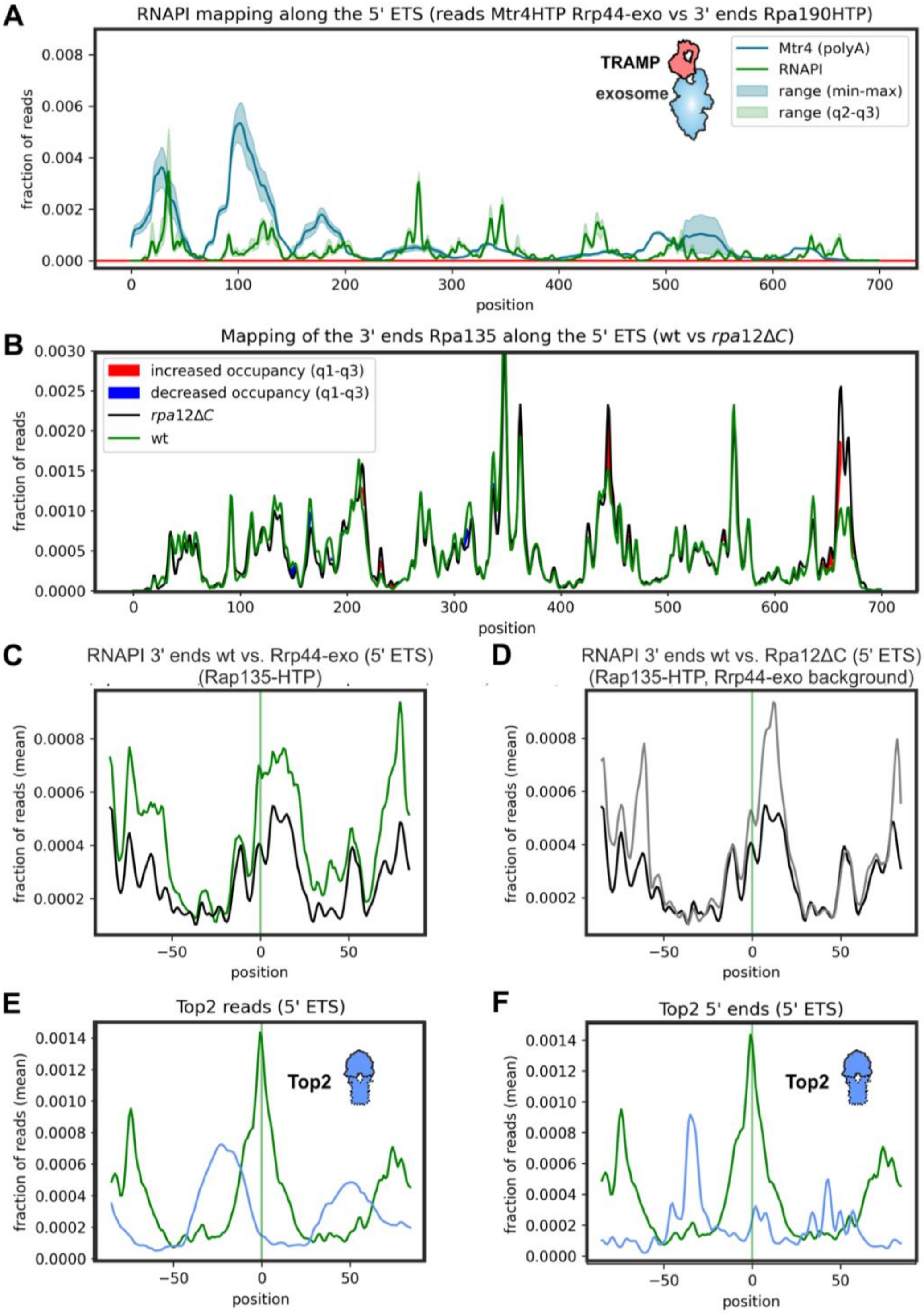


**Figure S5.** RNAPI and Top2 distributions along the 5' ETS (related to Figure 5).
A: Mtr4 (Rrp44-exo) total CRAC reads (blue) superimposed over Rpa190 3' end reads (green)s. Data are represented as mean (n=2).
B: Distribution of RNAPI (Rpa135-HTP) over the 5' ETS comparing WT and *rpa12ΔC* strains. Differences are highlighted in color. Data are represented as median (Rpa135-HTP n=7, Rpa135-HTP *rpa12ΔC* n=4).
C: RNAPI (Rpa135-HTP) peak metaplot for the 5' ETS, comparing WT (black) with *rrp44-exo (green)* strains
D: RNAPI (Rpa190-HTP) CRAC peak metaplot for the 5' ETS, comparing WT with *rrp44-exo* (black) or *rpa12ΔC* (gray) strains

E-F: Distribution of Top1-HTP CRAC (blue) over the 5’ ETS versus Rpa190 3’ ends (green) in a Rpa190 peak metaprofile: (E) total Top2 reads; (F) 5’ ends of Top2 reads

**Figure S6**

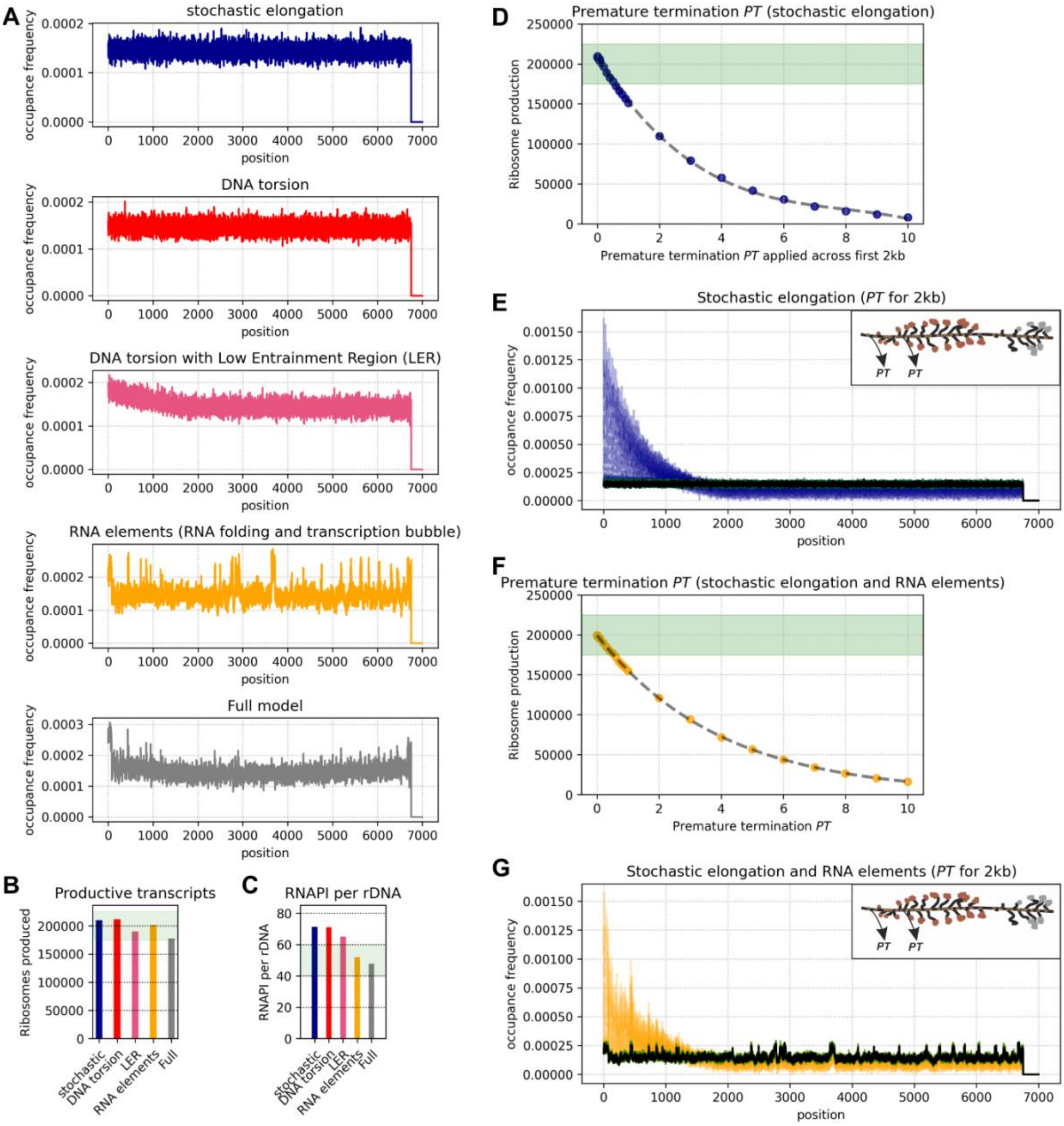


**Figure S6.** Development of premature termination $P_{PT}$ function (related to Figure 6)
A: Modeled RNAPI occupancy along the transcription unit using a model of stochastic initiation and discrete, stochastic elongation. The average RNAPI occupancy for 16 simulations is presented. Each simulation was run for 6,000 sec and 200 time points were collected.
B: Average number of productive transcription events for each type of simulation (corresponding to panel A).
C: The average number of RNAP molecules per transcription unit (corresponding to panel A).
D: $P_{PT}$ effect on ribosome production in model including stochastic elongation only. $P_{PT}$ was applied across the first 2kb of the transcription unit. Note that because $P_{PT}$ is applied only for this part of the transcription unit the values of $P_{PT}$ are significantly higher than for panel Fig 6A.
E: RNAPI occupancy profiles corresponding to panel D. Black – no premature termination $P_{PT}$, green $P_{PT}$ where ribosome synthesis is effective, blue – ribosome synthesis is decreased or insufficient.
F: $P_{PT}$ effect on ribosome production in model including stochastic elongation and RNA elements. Note that because $P_{PT}$ is applied only for the part of the transcription unit the values of $P_{PT}$ are significantly higher than for panel Fig. 6A.

G: RNAPI occupancy profiles corresponding to panel E. Black – no premature termination $P_{PT}$, green $P_{PT}$ where ribosome synthesis is effective, orange – ribosome synthesis is decreased or insufficient.

---

**Table S1**

| ID | Name | loc | sequence |
|---|---|---|---|
| PCR062 | RPA190-HTP tagging | fwd | ATGTTGGTACGGGTTCATTTGATGTGTTAGCAAAGGTT CCAAATGCGGCTGAGCACCATCACCATCACC |
| PCR063 | RPA190-HTP tagging | rev | ATGATGCACTTTATAATACCCTAGCTCTCTTAATTTACA TAACACACGCGTACGACTCACTATAGGG |
| PCR064 | Rpa190 check | fwd | TTTATCGACGTTGATGGTATTA |
| PCR065 | Rpa190 check | rev | GCATTGACAGGCTCCATAA |
| PCR073 | Rpa135-HTP tagging | fwd | CTGAGCTATCCGCAATGGGTATAAGATTGCGTTATAATG TAGAGCCCAAAGAGCACCATCACCATCACC |
| PCR074 | Rpa135-HTP tagging | rev | ACAATTGACCAAGCCTTCATTTACCATTCTATATCAATTTG GAAAGAAGGGTATACGACTCACTATAGGG |
| PCR075 | Rpa135 check | fwd | ATGTCGCGAGTGTGGTTCTATTTT |
| PCR076 | Rpa135 check | rev | AAGCCTGCACCTCTTGCAGTAGAA |
| EP98 | Nsi1 F1 deletion | fwd | CAAATTTTGTGCATAGAGCAAGCAGCCGTTTCTTGTCT GCGTCAAGAAGAAAGATAAAGGTAGACGGATCCCCGG GTTAATTAA |
| EP99 | Nsi1 R1 deletion | rev | TTTAAAAATCAGTAAATATGCTTTTATCTATTGGGTCTGT ATATGTTTGGGAAAGTAACCCTTCGAATTCGAGCTCGT TTAAAC |
| EP100 | Nsi1 test | fwd | GAGCTTTCCAAATGCGATA |
| JH1584 | MX4-6 rev | | TGCAGCGAGGAGCCGTAAT |
| EP97 | Rai1 TAPfwd | fwd | TCTCGGAATAACAAGCAAAGTCGGTATGACAATTCAAG AGCAAATAGGCGTTCCATGGAAAAGAGAAG |
| EP96 | Rai1Tap rev | rev | TTTATAAATTTGCGAAAACCTAAATTTACCATAAAATGC GCACGAGTAGTTTACGACTCACTATAGGG |
| PCR062 | Rpa190HIS | fw | ATGTTGGTACGGGTTCATTTGATGTGTTAGCAAAGGTT CCAAATGCGGCTGAGCACCATCACCATCACC |
| PCR063 | Rpa190TAP | rev | ATGATGCACTTTATAATACCCTAGCTCTCTTAATTTACA TAACACACGCGTACGACTCACTATAGGG |
| PCR255 | pMet-AID-6Flag-Rnt1 | fwd | TGCGCATATAGAAGAGAGCAAAACTGTCCTATTTACAA GCTTTTCAAAACAGACATGGAGGCCCAGAATA |
| PCR256 | pMet-AID-6Flag-Rnt1 | rev | GTTTATTATCATTCTGGGTTTTCTTTTTACCTGCTACTTT TGAGCCCATATCTGGCGCGCCACATCTAGA |
| PCR801 | RRP44_D551 CRISPR plasmid | fwd | GATCTCAATATCAACACATCCTGGGTTTTAGAGCTAG |
| PCR802 | RRP44_D551 CRISPR plasmid | rev | CTAGCTCTAAAACCCAGGATGTGTTGATATTGA |
| PCR805 | RRP44_D551 HDR | fwd | Gacaaaaagaaaggatctcagagataaacttatatgtagtatcgatcctcca ggCtgCgtCgatattAAT |
| PCR806 | RRP44_D551 HDR | rev | Acaccaacttcccaattaccgtttggaagctttttcgcatgtagggcat cATTaatatcGacGcaGcctg |

**Table S1.** Oligonucleotides used for strain construction.

**Table S2**

| ID | oligo | sequence |
|---|---|---|
| oTWT039 | 5'FAM | AGGCCGAAA |
| oTWT114 | | /56-FAM/rArGrArGrGrGrArUrUrA |
| oTWT150bio | | /5Biosg/ATACTTACAGCGTACCGATCACCCCCCCCCAGTAGTGA AGATTTTGTAATTAGTAGTAGTGAAGATTTTGGGTGGTAGAGGG AATAATCCCTCTAGT |
| oTWT151 | | TCCCTCTACCACCCAAAATCTTCACTACTACTAATTACAAAATCT TCACTACTGGGGGGGGGTGATCGGTAC |
| oTWT164 | | rUrCrUrArCrGrUrArUrArArGrArGrGrGrArUrUrA |
| oTWT165 | | rUrArUrArUrCrUrUrGrUrCrArArUrCrArUrArCrCrArGrArGrGrGrArUrUrA |

**Table S2.** Oligonucleotides used for *in vitro* assays.
